# *Fusobacterium nucleatum* Accelerates Atherosclerosis *via* Macrophage-Driven Aberrant Proinflammatory Response and Lipid Metabolism

**DOI:** 10.3389/fmicb.2022.798685

**Published:** 2022-03-11

**Authors:** Jieyu Zhou, Lin Liu, Peiyao Wu, Lei Zhao, Yafei Wu

**Affiliations:** ^1^State Key Laboratory of Oral Diseases, National Clinical Research Center for Oral Diseases, West China Hospital of Stomatology, Sichuan University, Chengdu, China; ^2^Department of Periodontics, West China Hospital of Stomatology, Sichuan University, Chengdu, China

**Keywords:** periodontitis, *F. nucleatum*, atherosclerosis, macrophage, polarization, inflammation, microRNA, lipid metabolism

## Abstract

Periodontitis, an oral chronic inflammatory disease, is reported to show an association with atherosclerotic vascular disease. *Fusobacterium nucleatum* is an oral commensal bacterium that is abundantly implicated in various forms of periodontal diseases; however, its role in the pathogenesis of atherosclerosis is unclear. This study aimed to elucidate the underlying pathogenic mechanisms of atherosclerosis induced by *F. nucleatum* to provide new insight on the prevention and treatment of atherosclerosis. We used an animal model, that is, ApoE^–/–^ mice were infected with *F. nucleatum* by oral gavage, and *in vitro* co-culture models to assess the pathogenicity of *F. nucleatum.* The results indicate that *F. nucleatum* ATCC 25586 invaded aortic tissues and substantially increased the progression of atherosclerotic lesions. In addition, *F. nucleatum* changed plaque composition into a less-stable phenotype, characterized with increased subcutaneous macrophage infiltration, M1 polarization, lipid deposition, cell apoptosis, and reduced extracellular matrix and collagen content. The serum levels of pro-atherosclerotic factors, such as interleukin (IL)-6, IL-1β, tumor necrosis factor (TNF)-α, monocyte chemoattractant protein-1 (MCP-1), c-reactive protein, and oxidized low-density lipoprotein (ox-LDL) and microRNAs (miR-146a, miR-155, and miR-23b) were considerably increased after *F. nucleatum* stimulation, whereas HDL-c level was reduced. *F. nucleatum* induced *in vitro* macrophage apoptosis in a time- and dose-dependent manner. *F. nucleatum* facilitated ox-LDL–induced cholesterol phagocytosis and accumulation by regulating the expression of lipid metabolism-related genes (AR-A1, ACAT1, ABCA1, and ABCG1). *F. nucleatum* further worsened the atherosclerotic plaque microenvironment by considerably increasing the levels of IL-6; IL-1β; TNF-α; MCP-1; and MMP-2, 8, and 9 and by suppressing fibronectin (FN) 1 levels during foam cell formation. This study shows that *F. nucleatum* ATCC 25586 is implicated in atherosclerosis by causing aberrant activation and lipid metabolism in macrophage.

## Graphical Abstract

Schematic diagram depicting the process of *F. nucleatum* inducing atherosclerosis. As one of the oral commensal bacteria, *F. nucleatum* is abundant in periodontitis sites and can enter blood circulation through damaged periodontal tissue. Subsequently, there is an increase in risk factors (dyslipidemia and inflammatory mediators) in the blood circulation. In addition, it has high invasiveness and can invade into the vascular intima, promoting macrophages M1 polarization, aberrant lipid metabolism, and cell apoptosis, thereby increasing the release of inflammatory mediators, and the degradation of extracellular matrix in the atherosclerotic plaque microenvironment. By then, plaques are prone to rupture.

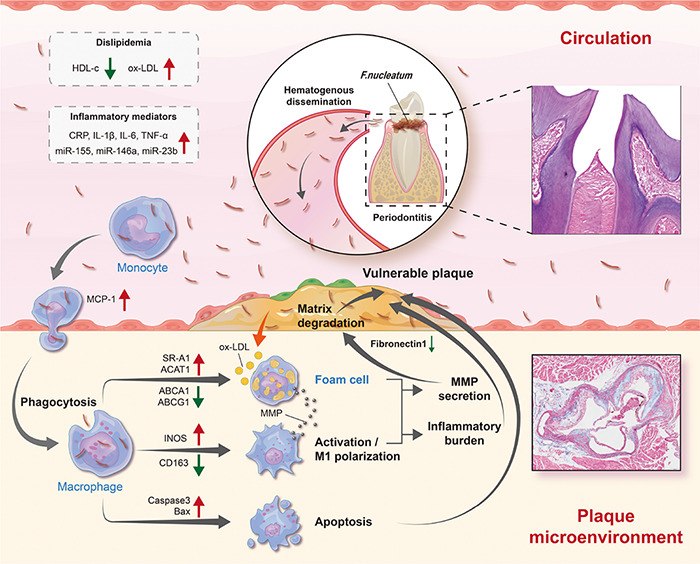

## Introduction

Atherosclerosis is a chronic inflammatory vascular disorder that occurs due to increased environmental risk factors, such as bacterial infection and systemic inflammatory states (Libby et al., 2018). Periodontitis is a common oral chronic inflammatory disease caused by microorganisms and is an independent risk factor for cardiovascular disease as reported in the consensus report of joint EFP/AAP (Tonetti et al., 2013). *Fusobacterium nucleatum* (*F. nucleatum*, Fn) is one of the most abundant species in the oral cavity, often acting in a synergistic pathogenic role during the process of periodontitis (Chukkapalli et al., 2015, 2017). Recent studies report that *F. nucleatum* exhibits strong virulence through adhesion and invasion to be one of the most prevalent oral species found in extraoral sites under disease conditions and involved in various extraoral infections and systemic disease processes, such as inflammatory bowel disease, colorectal cancer, and rheumatoid arthritis (Ismail et al., 2012; Han and Wang, 2013; Ebbers et al., 2018; Kashani et al., 2020).

Notably, the pathogenic mechanisms of *F. nucleatum* in atherosclerosis have not been fully uncovered to date. Only two animal studies report the direct effects of *F. nucleatum* on atherosclerosis, but these studies report contrasting results. Lee et al. (2012) report that intraperitoneal injection of heat-killed *F. nucleatum* ATCC 25586 and its heat-shock protein GroEL cause endothelial dysfunction and accelerate the progression of atherosclerotic lesions by inducing autoimmune responses (Lee et al., 2012). In contrast, another study reports that *F. nucleatum* ATCC49256 might play a protective role in atherosclerosis with evidence of the remission of atherosclerotic plaques after periodontal *F. nucleatum* infection (Velsko et al., 2015). Interestingly, we recently discovered that *F. nucleatum* ATCC 25586, could induce endothelial cell apoptosis and further stimulate THP-1 cell adhesion and transmigration in HUVECs, which suggests that *F. nucleatum* ATCC 25586 played a pathogenic role during atherosclerosis through destroying endothelial integrity and enhancing vascular permeability (Wang et al., 2019). There is a plethora of factors, such as cytokines, oxidized low-density lipoproteins, and cell debris in the atherosclerotic plaque microenvironment, and many studies shed light on the different phenotypes and functions that macrophages can acquire upon exposure to these external stimuli (Willemsen and de Winther, 2020). In advanced plaques, macrophages become the most abundant immune cell type and play a crucial role in atherosclerosis development (Kavurma et al., 2017). Thus, analyzing macrophage conditions under periodontal pathogen attack is important to reveal the association between periodontitis and atherosclerosis.

Recently, microRNAs are reported to be crucial regulatory molecules in atherosclerosis and endowed with significance in cardiovascular diseases as biomarkers (Feinberg and Moore, 2016). Serum miR-146a level was markedly increased in periodontitis patients, positively correlated with inflammatory cytokine levels, and considered as a key molecule associating periodontitis with acute coronary syndrome (Bagavad Gita et al., 2019). It is also reported to be significantly upregulated in atherosclerotic plaques, resulting in the formation of vulnerable plaque and disease progression (Raitoharju et al., 2011; Kin et al., 2012; Cao et al., 2015; Cheng et al., 2017). miR-155 is an important molecule that regulates the inflammatory signaling pathway of AS (Wei et al., 2013; Li et al., 2016). It is found that the expression of miR-155 in the plaques or plasma of AS patients and mice are significantly increased and are accompanied by the dysregulation of various inflammatory factors (Zhang and Wu, 2014; Li et al., 2016; Ma et al., 2017). miR-23b was reported to be significantly upregulated in coronary artery disease (CAD) when compared with the control group, suggesting the potential of miR-23b as a new biomarker in CAD diagnosis (Di et al., 2015). Some studies report that periodontal pathogens often affect the expression of microRNAs systematically (Nahid et al., 2011; Tomofuji et al., 2016; Xuan et al., 2017; Yoneda et al., 2019), and these microRNAs are closely related to the process of atherosclerosis, which makes it possible for microRNAs to be mediators bridging periodontal pathogens and atherosclerosis. A case-control study reported a difference in the serum microRNA level between periodontitis patients and healthy controls, and these microRNAs were considered as candidate serum biomarkers for chronic periodontitis (Yoneda et al., 2019). Experimental periodontitis also altered specific serum microRNA levels in wistar rats, and these microRNAs were suggested as valuable biomarkers for periodontitis. Thus, we hypothesized that microRNAs might act as biomarkers and regulators of atherosclerosis during *F. nucleatum* infection.

Overall, the effect and molecular mechanism of *F. nucleatum* on the atherosclerotic plaque microenvironment in atherosclerosis are unclear. In this study, we investigated the pro-atherosclerotic role of *F. nucleatum* ATCC 25586 using ApoE^–/–^ mice based on our previous research (Wang et al., 2019). Furthermore, we evaluated the detrimental effects of *F. nucleatum* on the activation, polarization, inflammation, apoptosis, matrix degradation, and cholesterol metabolism of macrophages in the atherosclerotic plaque microenvironment. Our study expects to provide a clear perspective for the association between periodontitis and atherosclerosis. Of note, our study is the first to evaluate the effect of *F. nucleatum* on the phenotypic transformation of macrophages and foam cells during atherosclerosis.

## Materials and Methods

Detailed methodology is available in Supplementary Material.

### Animals and Oral Infection Model

*F. nucleatum* ATCC 25586 was cultured anaerobically on blood agar plates (BD Biosciences, CA, United States) at 37°C for 2–6 days, followed by culturing in brain heart infusion broth (BD Biosciences, CA, United States) for 2 days. *F. nucleatum* cultured in the logarithmic growth phase was harvested by centrifugation at 4,000 rpm for 10 min and washed three times with phosphate buffered saline (PBS, Hyclone, United States). The bacterial concentration was spectrophotometrically standardized to optical density (OD) value at 540 nm = 0.8 *F. nucleatum* by using a microplate reader (Thermo Scientific, United States), corresponding to 1 × 10^9^ bacteria/ml (Wang et al., 2019). Then, the bacterial suspensions were centrifuged and diluted by 4% carboxymethyl cellulose (CMC)-PBS (Sigma, NY, United States), reaching the concentration of 1 × 10^9^ bacteria/ml. Six-week-old male ApoE^–/–^ mice, purchased from Beijing Vital River Laboratory Animal Technology Company, were housed in a specific pathogen-free controlled animal laboratory under 12-h dark/light photophase and fed with a standard chow diet to eliminate the effects of high cholesterol (Jawien, 2012; Rivera et al., 2013). Before oral inoculation, all mice (*n* = 16 in total) received sulfamethoxazole and trimethoprim daily mixed in the drinking water, and concurrently, oral cavities were rinsed with 0.12% chlorhexidine gluconate for 10 days to inhibit the colonization of other microorganisms, followed by a 3-day antibiotic wash-out period (Nahid et al., 2011). Next, the mice were randomly assigned to two groups: the infected group received oral inoculation with 100 μL of *F. nucleatum* suspended in 4% CMC-PBS (1 × 10^9^ bacteria ml^–1^), and the sham-infected group received the same volume of 4% CMC-PBS as sterile vehicle (*n* = 8 in each group) (Velsko et al., 2015). The oral inoculation was administered every alternate day for 12 weeks. All animal procedures were conducted in conformance to the guidelines recommended by Directive 2010/63/EU of the European Parliament regarding the protection of animals employed for scientific purposes and approved by the Ethics Committee of Sichuan University (No. WCHSIRB-D-2017-071).

All mice were sacrificed by exsanguination under anesthesia with 1.2% tribromoethanol (Avertin; Macklin, Shanghai; 240 mg/Kg; intraperitoneal injection). The right maxillary and mandible specimens were prepared for histological examination. The left maxillae and mandibles were scanned using a high-resolution micro-CT scanner. The atherosclerotic plaque features and monocyte recruitment were assessed by H&E staining, Oil Red O staining, Masson staining, and immunofluorescence staining. The bacterial load in the aortic tissue was quantified by absolute qPCR. Detailed methodology of assays is exhibited in the Supplementary Materials.

### Cell Culture and Co-culture Model

Human monocyte cell line THP-1 (TIB-202) cells were purchased from the American Type Culture Collection (ATCC) and cultured in RPMI1640 medium (Gibco, United States) supplemented with 10% FBS (Gibco, United States) and 1% 100 U/ml penicillin–streptomycin (Gibco, United States) under a 5% CO_2_ atmosphere at 37°C. THP-1 monocytes were differentiated into macrophages (THP-1-derived macrophages, dTHP1) with the treatment of 100-nM PMA (Sigma-Aldrich, United States) for 48 h. The antibiotics were removed from the medium before co-culturing the bacterial and host cells. *F. nucleatum* was centrifuged and suspended in the RPMI 1640 (Gibco, United States). After the concentration was measured by using a microplate reader (Wang et al., 2019), the bacterial suspensions were added into dTHP1 cells at different multiplicity of infection (MOI) bacteria:cells of 0, 10, 100, 200, and 500, followed by co-culturing to the indicated time points (24 and 48 h) under a humidified 5% CO_2_ atmosphere at 37°C. Intracellular bacteria and phagocytotic particles were observed through confocal microscopy. Macrophage apoptosis and phagocytosis were quantified by flow cytometry. Foam cell formation was detected with Oil Red O staining. The cytokine levels were assessed by using ELISA kits. The expression of genes and microRNAs associated with atherosclerosis was assessed by qRT-PCR. Detailed methodology of assays is exhibited in the Supplementary Materials.

### Statistical Analyses

All statistical data were analyzed by using the SPSS version 21.0 software. Comparisons between the groups were performed using Student’s *t*-test. For multiple comparisons, the data were analyzed by one-way analysis of variance (ANOVA) with *post hoc* Tukey. Spearman correlation analysis was conducted to assess the correlation between the extent of atherosclerotic lesion and the serum microRNA levels. The data were characterized as mean ± SD of at least three independent experiments unless otherwise specified. *P* < 0.05 was considered to be statistically significant.

## Results

### *Fusobacterium nucleatum* Induces Alveolar Bone Resorption, Aorta Innate Immune Response, and Vulnerable Plaque Formation

**FIGURE 1:**
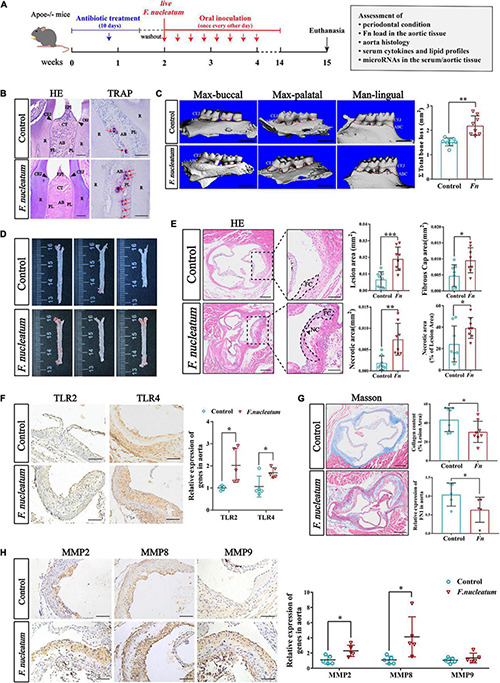
Evaluation of periodontal condition and atherogenesis induced by oral *F. nucleatum* infection. **(A)** The animal experimental setup. **(B)** H&E staining and TRAP staining indicated the interproximal areas between the first and second mandible molars. The red arrows revealed TRAP-positive multinucleated osteoclasts. CEJ, cementum-enamel junction; CT, connective tissue; R, root; EPI, oral epithelium; PL, periodontal ligament; AB, alveolar bone. Scale bars, 100 μm. **(C)** The images show the reconstructed three-dimensional images from the computerized tomography of the right maxilla buccal, maxilla palatal, and mandible lingual sides. More bone loss and root bifurcation exposure could be observed in the infected group compared with the control group. Scale bar, 1 mm. The area between the cemento-enamel junction (CEJ) and the alveolar bone crest (ABC) of molars 1, 2, and 3 of maxilla palatal, maxilla buccal, and mandible lingual sides was measured to calculate the total ABR. The infected group had statistically increased alveolar bone resorption relative to the control group (*n* = 8, each group). **(D)** Atherosclerotic lesion areas in *en face* aortas were assessed by Oil Red O staining. **(E)** The left panels showed the representative histological analysis of aortic sinus cross-sections stained with H&E. Scale bars, 200 μm. Enlarged images from the black-framed areas in the right panels revealed how the necrotic core area and fibrous cap were defined for quantification in each section. Dashed lines show the boundary of the developing necrotic core (NC) and fibrous cap (FC). The black arrow showed the cholesterol crystals in plaques. Scale bars, 100 μm. Scatterplots are the mean lesion area calculated from aortic sinus H&E cross-sections as well as the quantification of the necrotic core area and fibrous cap. Each dot represents the mean of the quantification of five sections from an individual animal (*n* = 8, each group). **(F)** Immunohistochemistry staining of TLR2 and TLR4 proteins; Scale bars, 100 μm. qRT-PCR analyze the relative mRNA levels of TLR2 and TLR4 in aortic tissues (*n* = 5, each group). **(G)** Masson staining shows the representative aortic sinus cross–sectional analysis of collagen content. Scale bars, 200 μm. Scatterplots exhibit quantifications of collagen content expressed as the average percentage of collagen per total lesion area. (*n* = 8, each group). **(H)** Immunohistochemistry staining of matrix metalloproteinases (MMP2, MMP8, and MMP9); Scale bars, 100 μm. Data are expressed as the relative mRNA levels of the matrix metalloproteinases in the aortic tissues (*n* = 5, each group). All data represent the mean ± SD. *p < 0.05, ^**^p < 0.01, ^***^p < 0.001 vs. control group Student’s *t*-test.

To evaluate the effect of *F. nucleatum* on the cardiovascular system, we established atherosclerosis models by using ApoE^–/–^ mice (Jawien, 2012). The infected group received oral inoculation with 100 ul *F. nucleatum* once every alternate day, and the control group received vehicle (PBS-CMC) only (Velsko et al., 2015). As shown in the flow chart (Figure 1A), the course of infection lasted for 12 weeks. During the experiment, the body weight of mice in both groups increased steadily with no significant difference in body weight gain between the two groups (*p* > 0.05) (Supplementary Figure 1). H&E staining of maxilla tissue sections showed that there was mild epithelial hyperplasia, loose collagen fibers, and slight gingival inflammation with no significant inflammatory cell infiltration in the connective tissue of the infected group (Figure 1B, the left panel). *F. nucleatum* also increased the immunostaining for proinflammatory cytokines IL-6, IL-1β, IL-17, and TNF-α in gingival compared with the control group (Supplementary Figure 2). TRAP staining indicated that more osteoclasts were distributed on the surface of the alveolar bone in the infected group (Figure 1B, the right panel, red arrows). From the 3-D reconstruction images, we observed significant alveolar bone resorption and root bifurcation exposure in the infected group (Figure 1C). Micro-CT quantitative analysis revealed that there was statistically significant alveolar bone resorption in the infected group as compared with the control group (*p* < 0.01) (Figure 1C). These results indicated that *F. nucleatum* induced mild inflammatory infiltration and significant bone resorption.

Absolute qPCR analysis showed that the copies of *F. nucleatum* 16S rDNA could be detected in higher numbers in the aortic tissues of infected mice (*p* < 0.05) (Supplementary Figure 4). Sequencing of qRT-PCR products showed 98–100% homology with the standard *F. nucleatum* ATCC 25586 sequence from NCBI GenBank. The sequences of qRT-PCR products are listed in Supplementary Table 3. The results suggest that the *F. nucleatum* DNA detected in aortic tissues was a consequence of oral inoculation of the bacterium and also indicate that *F. nucleatum* has the ability of systemic transmission and invasion to aortic tissue.

To investigate the effect of *F. nucleatum* on the development of atherosclerosis, aortic lesion areas were assessed using two independent methods, the en face morphometric analysis of the entire aortic tree and paraffin sections analysis of the aortic sinus. From Oil Red O staining of the en face aortas, we observed that the infected group developed larger lesion areas than the control group (Figure 1D). Considering that the area of the aortic sinus is extremely susceptible to atherosclerosis, we evaluated the lesion areas in the aortic sinus cross-section using H&E staining. A significant increase in plaque areas of the infected group was observed as compared with the control group (*p* < 0.001) (Figure 1E). In addition, the areas of plaque necrosis and fibrous caps were also found to be larger in the infected group (*p* < 0.05) (Figure 1E). To further evaluate the plaque vulnerability, the proportion of necrotic area in the lesion was calculated and also found to be significantly enhanced in the infected group (*p* < 0.05) (Figure 1E). Next, the effect of *F. nucleatum* on innate immune response during the invasion process was assessed. Expression of toll-like receptors in aortic tissues was detected by immunohistochemistry and qRT-PCR. Immunohistochemical analysis as well as qRT-PCR (*p* < 0.05), showed higher expression of TLR2 and TLR4 in the infected group (*p* < 0.05) (Figure 1F). We also analyzed whether *F. nucleatum* influenced the extracellular matrix in lesions since it is associated with plaque stability. The content of collagen and FN1 in plaques of the infected group was lower compared with the control group (*p* < 0.05) (Figure 1G). Immunohistochemistry also showed that the matrix metalloproteinases (MMP2, MMP8, MMP9), which cause collagen degradation, were higher in the infected group by immunohistochemistry. Particularly, MMP2 and MMP8 levels exhibited a significant increase in expression (*p* < 0.05) (Figure 1H).

### Enhanced Monocyte/Macrophage Recruitment, M1 Activation, Foam Cell Formation, and Apoptosis in the Aorta Wall Under *F. nucleatum* Infection

**FIGURE 2:**
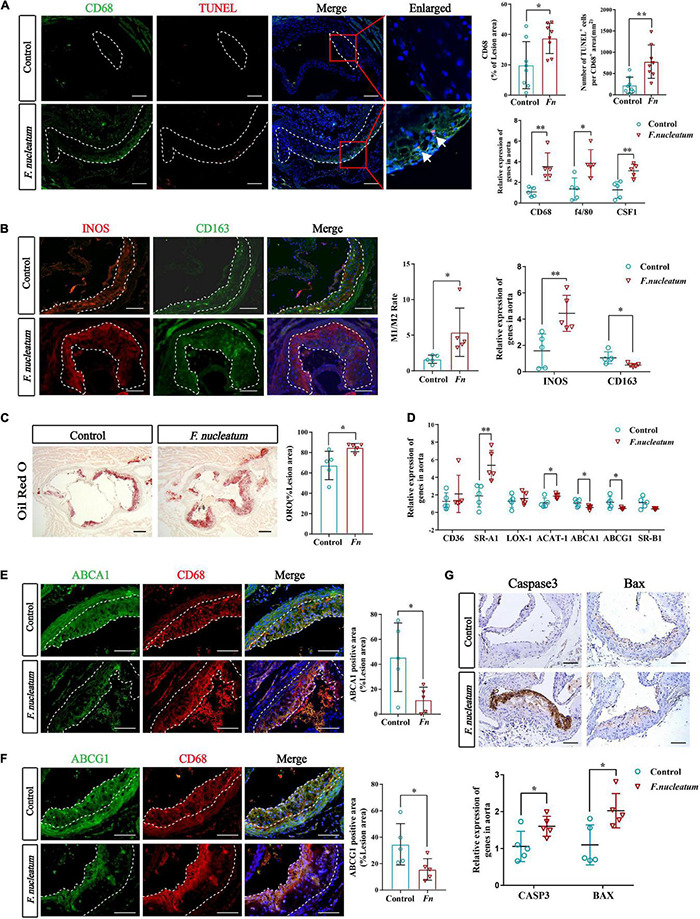
*F. nucleatum* promotes monocytes/macrophage accumulation, polarization, foam cell formation, and apoptosis in the plaque microenvironment. **(A)** The left panel shows representative aortic sinus cross–sectional analysis for macrophage content *via* immunodetection of CD68 and apoptotic cell *via* TUNEL staining (the white arrows in enlarged panels). Dashed lines show the boundary of the plaques. Scale bars, 100 μm. Data are expressed as the average percentage of CD68–positive in the lesion area, and the number of TUNEL–positive cells per CD68–positive area (*n* = 8, each group) in the right panel. The relative mRNA levels of cell markers for macrophages were obtained after normalization with GAPDH by qRT-PCR. **(B)** Immunofluorescence staining labels the macrophage polarization markers (INOS and CD163) in the left panel; Dashed lines show the boundary of the plaques. Scale bars, 100 μm. The M1/M2 macrophage ratio was calculated from their positive areas (*n* = 5, each group). The relative mRNA levels of INOS and CD163 in the aortic tissues were analyzed by qRT-PCR (*n* = 5, each group). **(C)** Lipid droplets in foam cells were visualized by Oil Red O (ORO) staining; Scale bars, 100 μm. Data are expressed as the proportion of ORO–positive area (*n* = 5, each group). **(D)** The relative expression of cholesterol metabolism-related genes in the aortic tissues (*n* = 5, each group). **(E,F)** Immunofluorescence staining label the cholesterol metabolism-related proteins ABCA1 and ABCG1 (green). Dashed lines show the boundary of the plaques. ABCA1 and ABCG1 immunoreactivity was determined by color and normalized to lesion size (*n* = 5, each group); Scale bars,100 μm. **(G)** Immunohistochemistry stains apoptosis protein; Scale bars, 100 μm. Data are expressed as the relative mRNA levels of CASP3 and BAX in the aortic tissues (*n* = 5, each group). All data represent the mean ± SD. *p < 0.05, ^**^p < 0.01 vs. control group. Student’s *t*-test.

The presence of cholesterol-laden macrophages or foam cells in the subintima of arteries is the prominent feature of atherosclerosis (Yu et al., 2013). A significant increase in the proportion of the CD68-positive area in plaques of the infected group (*p* < 0.05) was observed from the immunofluorescence analysis (Figure 2A). The macrophage markers CD68 and F4/80, along with colony-stimulating factor 1 (CSF1) showed significantly higher expression in the infected group (*p* < 0.05) (Figure 2A). In addition, the number of TUNEL-positive cells (labeled apoptotic cells) per CD68-positive area was also elevated in the infected group (*p* < 0.01) (Figure 2A). We found that the ratio of M1 to M2 macrophages in the aortic wall was also increased (*p* < 0.05) (Figure 2B). Similarly, the M1 polarization marker INOS was significantly upregulated in the infected group (*p* < 0.01), and the M2 polarization marker CD163 was downregulated (*p* < 0.05) (Figure 2B).

We evaluated the lipid deposition and foam cell formation in plaques *via* Oil Red O staining. The proportion of Oil Red O positive area in plaques of the infected group was larger than that of the control group (*p* < 0.05) (Figure 2C). We investigated the expression of cholesterol metabolism-related genes in aortic tissues and the results indicated that the imbalance of cholesterol metabolism in macrophages promotes foam cell formation. The genes associated with cholesterol uptake, like Scavenger receptor A1 (SR-A1), were significantly upregulated in the infected group (*p* < 0.01), whereas Cluster Determinant 36 (CD36) and lectin−like ox-LDL receptor 1 (LOX-1) did not show a statistically significant difference in expression (Figure 2D). Cholesterol acyltransferase 1 (ACAT-1), the gene that mediates cholesterol transformation in macrophages, was upregulated in the infected group (*p* < 0.05) (Figure 2D). On the contrary, the expression of ATP-binding cassette transporter A1 (ABCA1) and ATP-binding cassette transporter G1 (ABCG1), involved in cholesterol efflux, were downregulated (*p* < 0.05), and scavenger receptor B1 (SR-B1) levels showed no significant change (Figure 2D). Immunofluorescence analysis also indicated the prominent decrease in expression of Abca1 and Abcg1 in atherosclerotic plaques of infected mice (Figures 2E,F).

The expression of apoptosis-related protein markers such as Caspase3 and Bax, was analyzed in the lesions by immunohistochemical staining and qRT-PCR. We found higher levels of Caspase3 and Bax in the aortas of the infected group (*p* < 0.05) (Figure 2G). Overall, these results demonstrate that *F. nucleatum* could promote macrophage infiltration, M1 skewing, foam cell formation, and apoptosis, which further resulted in deterioration of the plaque microenvironment.

### *Fusobacterium nucleatum* Induces Dyslipidemia and Elevated Pro-atherosclerotic Cytokines *in vivo*

**FIGURE 3:**
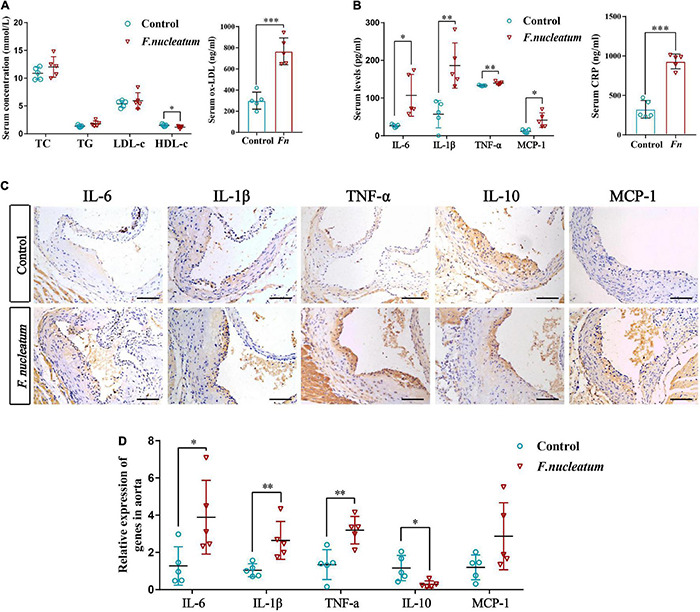
*F. nucleatum* alters serum lipid profiles and systemic cytokine levels. **(A)** Serum levels of total cholesterol (TC), total triglyceride (TG), high-density lipoprotein cholesterol (HDL-c), low-density lipoprotein cholesterol (LDL-c), and oxidized low-density lipoprotein (ox-LDL) (*n* = 5, each group). **(B)** Serum levels of proinflammatory cytokines TNF-α, IL-1β, IL-6, CRP, and chemokine MCP-1 (*n* = 5, each group). **(C)** Inflammatory mediators in the aortic tissues are shown by immunohistochemical staining; Scale bars, 100 μm. **(D)** Data are expressed as the relative mRNA levels in the aortic tissues (*n* = 5, each group). All data represent the mean ± SD. *p < 0.05, ^**^p < 0.01 vs. control group. Student’s *t*-test.

Both dyslipidemia and inflammation are risk factors involved in the pathology of atherosclerosis. Investigation of the effects of *F. nucleatum* on lipid profiles indicated non-significant differences in TC, TG, and LDL-c between the infected and control groups. However, serum HDL-c levels in the infected group were lower than that in the control group (*p* < 0.05) (Figure 3A). Conversely, *F. nucleatum* significantly upregulated serum ox-LDL levels (*p* < 0.001) (Figure 3A). ELISA was performed to detect the pro-atherosclerotic mediators in serum to further analyze the levels of systemic inflammation. In comparison to the control group, infected mice showed higher levels of proinflammatory cytokines (TNF-α, IL-1β, IL-6, and CRP) and chemokine MCP-1 in serum (*p* < 0.05) (Figure 3B).

The increasing pro-atherogenic cytokines in the plaque microenvironment could contribute to the development of atherosclerosis (Shafi, 2020). To examine the pro-atherogenic mediators during *F. nucleatum* challenge, protein levels in the aorta were detected by immunohistochemistry. We observed elevated levels of proinflammatory cytokines (IL-6, IL-1β, TNF-α, and MCP-1) and decreased levels of anti-inflammatory mediator IL-10 in the infected group by immunohistochemical staining (Figure 3C). Similarly, the qRT-PCR analysis revealed higher levels of proinflammatory mediators IL-6, IL-1β, and TNF-α (*p* < 0.05) and lower levels of IL-10 (*p* < 0.05) in the infected group as compared with the control group (Figure 3D). The increase in the levels of MCP-1 was not statistically significant (Figure 3D).

### Effects of *Fusobacterium nucleatum* on Systemic MicroRNA Levels

**FIGURE 4:**
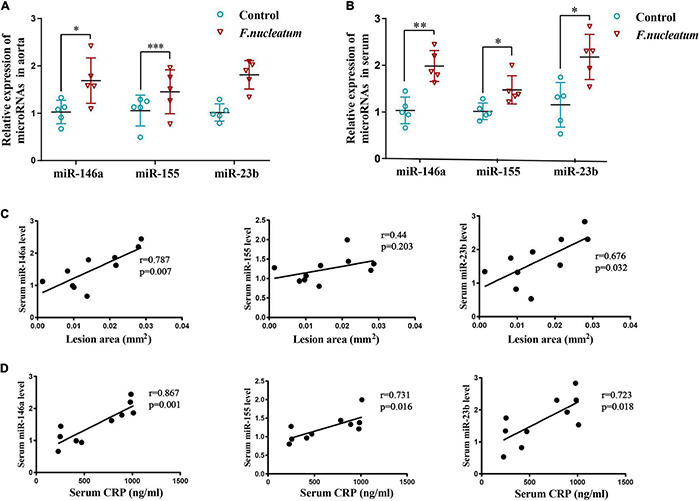
*F. nucleatum* alters microRNA levels in serum and aortas. **(A)** MicroRNAs related to atherosclerosis (miR-146a, miR-155, miR-23b) in the aortic tissues (*n* = 5, each group), normalized with U6. **(B)** Serum levels of microRNAs (miR-146a, miR-155, miR-23b) related with atherosclerosis, normalized with exogenous miRNA (CR100-01, Tiangen, China) (*n* = 5, each group). Values represent the mean ± SD. *p < 0.05, ^**^p < 0.01, ^***^p < 0.001 vs. control group. Student’s *t*-test. **(C)** Spearman correlation analysis was performed to evaluate the association between serum microRNAs (miR-146a, miR-155, miR-23b) levels and atherosclerotic lesion areas (*n* = 5, each group). **(D)** Spearman correlation analysis was performed to evaluate the association between serum microRNAs (miR-146a, miR-155, miR-23b) levels and CRP levels (*n* = 5, each group).

The expression of miR-146a and miR-23b was markedly enhanced in the aortic tissue of the infected group relative to the control group (*p* < 0.05) (Figure 4A). The level of miR-155 showed only a slight increase, which was not statistically significant (*p* > 0.05) (Figure 4A).

Micro-RNAs present in serum during *F. nucleatum* infection were also identified. The levels of serum miR-146a, miR-155, and miR-23b were significantly upregulated in the infected group (*p* < 0.05) (Figure 4B). To study the correlation between microRNAs induced by *F. nucleatum* and atherosclerosis, a correlation analysis was conducted for the serum miR-146a, miR-155, and miR-23b levels with plaque areas and the serum CRP levels, respectively. The levels of miR-146a (*r* = 0.787, *p* < 0.05) and miR-23b (*r* = 0.676, *p* < 0.05) and plaque areas showed strong positive correlation, and serum miR-155 levels had no significant correlation with plaque areas (*r* = 0.44, *p* > 0.05) (Figure 4C). Further, the correlation between miRNA levels and levels of inflammation was analyzed, and it showed that the serum levels of miR-146a, miR-155, and miR-23b were correlated positively with the serum CRP levels (*r* = 0.867, *r* = 0.731, *r* = 0.723, respectively, *p* < 0.05) (Figure 4D). These results indicate that the serum levels of miR-146a, miR-155, and miR-23b serve as inflammatory markers, and miR-146a, miR-23b could predict the severity of atherosclerosis during *F. nucleatum* infection.

### The Invasion of *Fusobacterium nucleatum* Promotes Macrophage Apoptosis in the Co-culture Model

**FIGURE 5:**
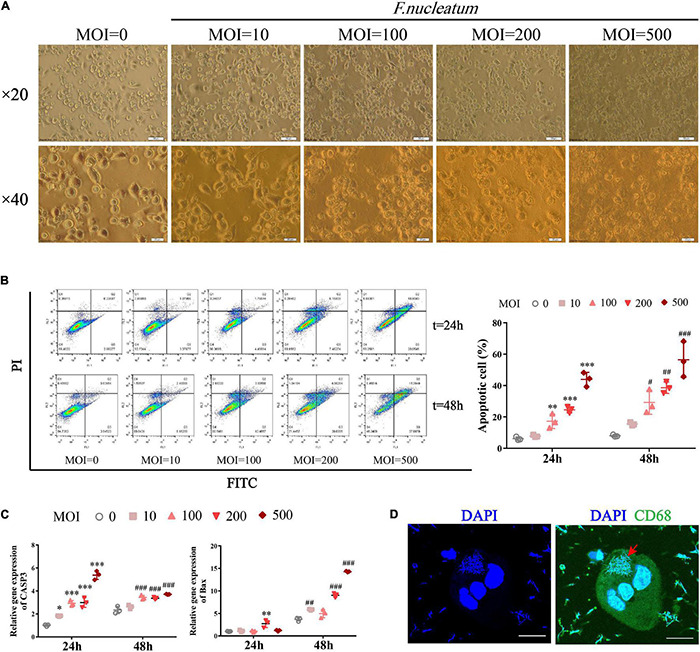
*F. nucleatum* can invade and induce macrophage apoptosis. To establish a co-culture model, dTHP1 were infected with *F. nucleatum* at different MOI of 0, 10, 100, 200, and 500 for 24 or 48 h, respectively. **(A)** Morphology of infected dTHP1 cells (× 20, × 40) was observed by a light microscope at 24 h. Scale bars, 50 and 20 μm. **(B)** The apoptotic rate of dTHP1 cells under different MOI were analyzed by flow cytometry at 24 and 48 h. **(C)** qRT-PCR analysis of apoptosis genes expression in dTHP1 cells at different MOI. The fold change of mRNA was calculated relative to the 24 h control group. **(D)** Immunofluorescence cross-section through dTHP1 cell after 24 h exposure to *F. nucleatum* (MOI = 100) was observed by a confocal laser scanning microscope (× 240). Scale bars, 10 μm. The red arrow indicates the bacterium in the cytoplasm. Data represent the mean ± SD of three duplicates. *p < 0.05, ^**^p < 0.01, ^***^p < 0.001 vs. control group (MOI = 0, 24 h); ^#^*p* < 0.05, ^##^*p* < 0.01, ^###^*p* < 0.001 vs. control group (MOI = 0, 48 h); The one-way ANOVA with Tukey’s *post-hoc* tests.

We investigated whether *F. nucleatum* influences the survival of macrophages *in vitro*. Differentiated THP1 cells were treated with bacteria at different MOI (0, 10, 100, 200, 500) for varied time intervals (24 and 48 h). The infected dTHP1 cells exhibited fusiform-like morphology (Figure 5A). Especially under high MOI (exceeding 100), some macrophages shrunk in size and foamed with a large number of secretory vesicles around, which is characteristic of the apoptotic process (Figure 5A; Elmore, 2007). The apoptotic rate of dTHP1 cells was found to be significantly increased at MOI of 100 or more after infection for 24 and 48 h, and the apoptotic rate increased in a dose-dependent manner through flow cytometry analysis (*p* < 0.05) (Figure 5B). Furthermore, the qRT-PCR analysis revealed that *F. nucleatum* induced markers showed increased expression in the apoptotic genes including *Caspase3* and *Bax* (*p* < 0.05) (Figure 5C). Large amounts of spindle-shaped rods of *F. nucleatum* were observed in the cytoplasm of dTHP1 cells after infection at the MOI of 100 for 24 h by confocal microscopy tomo-scanning (Figure 5D). These results indicate that *F. nucleatum* is strongly invasive, and they promote cell apoptosis at the high MOI (100 or more) in a dose- and time-dependent manner while the host cells could survive at a low MOI (10).

### *Fusobacterium nucleatum* Activates Macrophage Phagocytosis and Intracellular Lipid Accumulation and Further Accelerates Foam Cell Formation

**FIGURE 6:**
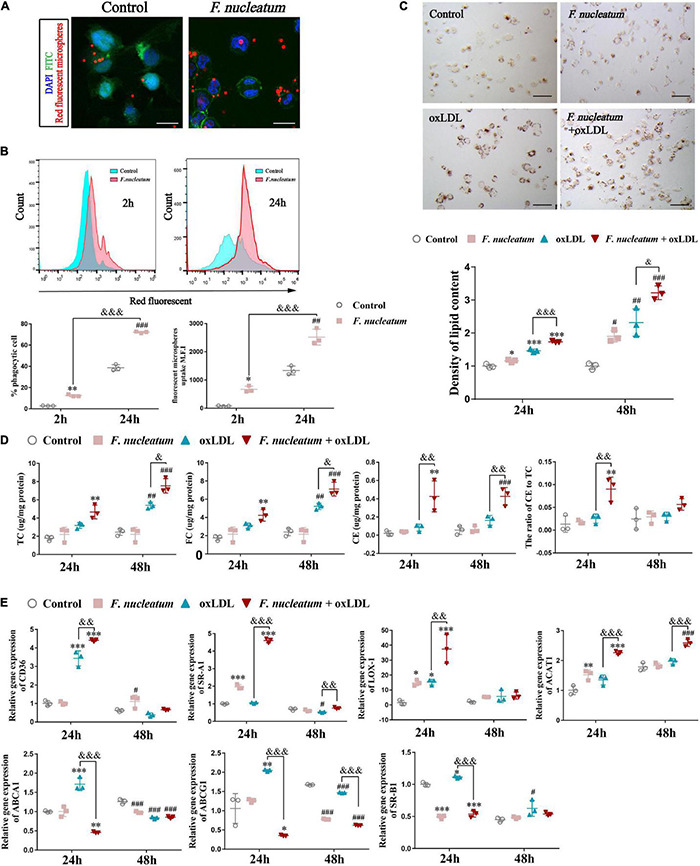
*F. nucleatum* promotes macrophage phagocytosis and foam cell formation. **(A)** Representative confocal microscopy image exhibited the engulfment of fluorescent microspheres by macrophages for 2 h. *F. nucleatum* group, after 24 h infection. control group, uninfected. Scale bars, 25 μm. **(B)** Flow cytometry analysis of 2 h fluorescent microspheres (50:1) uptake in macrophages after infection with *F. nucleatum* for 2 and 24 h at 37°C. The results were expressed as a percentage of positive phagocytosis and geometric mean fluorescence intensity (M.F.I.) after subtracting the autofluorescence of cells incubated in the absence of fluorescent microspheres. **(C)** Representative images of macrophages incubated with or without ox–LDL (50 μg/ml) for 24 h, then stained with ORO. Scale bars, 75 μm. The panel below shows the quantification of lipid droplets accumulated in the cytoplasm at 24 and 48 h, which were released using isopropyl alcohol and measured at 510 nm. Data were expressed as relative density to the control group. **(D)** Quantification of total cholesterol (TC), free cholesterol (FC), cholesterol esters (CE), and the ratio of CE to TC (CE/TC rate) in macrophages after treated as before. **(E)** The relative expression of genes involved in cholesterol metabolism after macrophages incubated with or without ox–LDL (50 μg/ml) for 24 and 48 h under infection/non-infection conditions. The fold change of mRNA was calculated relative to 24 h control group. Data represent the mean ± SD of three duplicates. *p < 0.05, ^**^p < 0.01, ^***^p < 0.001, vs. control group (24 h); ^#^*p* < 0.05, ^##^*p* < 0.01, ^###^*p* < 0.001 vs. control group (48 h); ^&^*p* < 0.05, ^&&^*p* < 0.01, ^&&&^*p* < 0.001, comparison among groups; The one-way ANOVA with Tukey’s *post-hoc* tests.

Differentiated THP1 cells were infected with *F. nucleatum* for 2 or 24 h, and then we removed the extracellular bacterium *via* vigorous washing. Next, we incubated the dTHP1 cells with red fluorescent microspheres for another 2 h to study whether *F. nucleatum* influenced the phagocytic capacity of macrophages. We observed the engulfment of fluorescent microspheres by macrophages using confocal microscopy (Figure 6A). Quantification by flow cytometry revealed that the rate of fluorescent positive cells and M.F.I. in the infected group increased significantly, and with infection at 24 h it is higher than that at 2 h (*p* < 0.05), indicating that fluorescent microspheres were efficiently engulfed by the infected cells relative to that by the uninfected cells (Figure 6B). In the plaque microenvironment, enhanced phagocytosis of macrophages resulted in excessive lipid intake. Lipid accumulation and foam cell formation are of great importance in the early stage of atherosclerotic lesions. Thus, we incubated dTHP1 cells in the presence of ox-LDL for 24 or 48 h to stimulate the foam cell formation. Oil Red O staining results showed red lipid droplets in the cytoplasm of ox-LDL-treated cells (Figure 6C). After alcohol extraction, we noted a significant increase in lipid accumulation of the ox-LDL treated cells, which was further aggravated by the presence of *F. nucleatum* at 24 and 48 h (*p* < 0.05) (Figure 6C). Consistent with the lipid content outcomes, we noted a significant increase in the TC, FC, and cholesterol esters (CE) at 48 h as well as the CE/TC rate at 24 h in the infected cells relative to those in the uninfected cells (*p* < 0.05) (Figure 6D).

We assessed the expression of cholesterol metabolism-related genes during *F. nucleatum* infection and foam cell formation to find the molecular mechanisms involved in cholesterol imbalance. Our results showed the expression of genes concerned with lipoprotein uptake, such as CD36, SR-A1, and LOX-1, were significantly upregulated at 24 h in *F. nucleatum* and the ox-LDL treated group when compared with that in the control group and the ox-LDL alone treatment group (*p* < 0.05) (Figure 6E). The expression of ACAT-1, which is responsible for esterifying FC into CE, was also upregulated significantly at 24 h in *F. nucleatum* and ox-LDL treated group (*p* < 0.05) (Figure 6E). In addition, the expression of ABCA1, ABCG1, and SR-B1, the genes regulating cholesterol efflux, were noted to reduce at 24 h in *F. nucleatum* and the ox-LDL treated group (*p* < 0.05) (Figure 6E). These results suggest that *F. nucleatum* could further aggravate the imbalance in cholesterol homeostasis disturbed by ox-LDL, accompanied with the dysregulation of cholesterol metabolism-related genes.

### Effects of *Fusobacterium nucleatum* on Macrophage Polarization and Pro-atherogenic Mediators During Foam Cell Formation

**FIGURE 7:**
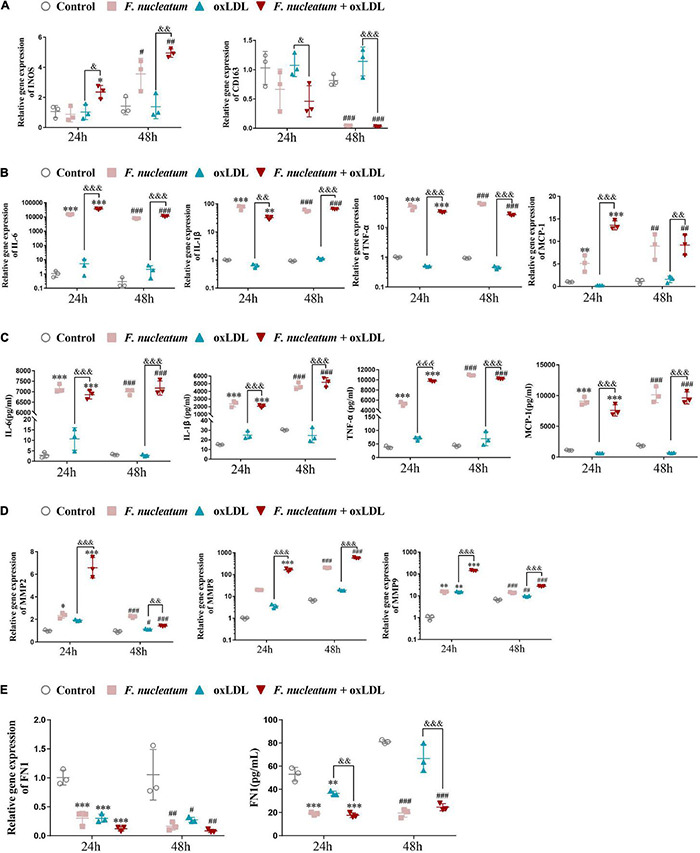
*F. nucleatum* infection induces M1 macrophage skewing, proinflammatory cytokines, and chemokine production. **(A)** M1-polarized phenotype marker (INOS) and M2-polarized phenotype marker (CD163) were assessed by qRT-PCR under 24 or 48 h infection at MOI of 100:1. **(B)** Pro-inflammatory cytokines (IL-1β, TNF-α, IL-6) and chemokine (MCP-1) were assessed by qRT-PCR. **(C)** Pro-inflammatory cytokines (IL-1β, TNF-α, IL-6) and chemokine (MCP-1) were assessed by ELISA. **(D)** Matrix metalloproteinases (MMP-2, MMP-8, MMP-9) were assessed by qRT-PCR. **(E)** Extracellular matrix (ECM) protein FN1 were assessed by qPCR and ELISA. The fold change of mRNA was calculated relative to 24 h control group. Data represent the mean ± SD of three duplicates. *p < 0.05, ^**^p < 0.01, ^***^p < 0.001 vs. control group (24 h). ^#^*p* < 0.05, ^##^*p* < 0.01, ^###^*p* < 0.001 vs. control group (48 h). ^&^*p* < 0.05, ^&&^*p* < 0.01, ^&&&^*p* < 0.001 comparison among groups; The one-way ANOVA with Tukey’s *post-hoc* tests.

After stimulation by microenvironmental factors in the plaque, macrophages exhibit different polarization states (Murray et al., 2014). In turn, the macrophage M1/M2 polarization balance determines the phenotype of plaques. As macrophage M1 skewing induces the secretion of proinflammatory factors and matrix metalloproteinase, the vulnerable plaque develops (Yang et al., 2020). The expression of genes regulating macrophage polarization was analyzed by qRT-PCR. Compared with the ox-LDL alone treatment group, the expression of M1-polarized maker INOS was highly induced (*p* < 0.05), and the expression of scavenger receptor CD163, an M2-polarized marker, exhibited significantly reduced in the *F. nucleatum* and ox-LDL treated group at 24 and 48 h (*p* < 0.05) (Figure 7A). The proinflammatory cytokines IL-6, IL-1β, TNF-α, and chemokine MCP-1 were all highly expressed after *F. nucleatum* infection for 24 or 48 h (Figure 7B). ELISA assay revealed consistent results with the qRT-PCR assay, suggesting that dTHP1 cells secreted large amounts of proinflammatory cytokines (IL-6, IL-1β, and TNF-α) and chemokine MCP-1 into the supernatant during the infection process (*p* < 0.05) (Figure 7C).

Next, we determined the effect of *F. nucleatum* on the extracellular matrix, associated with plaque stability. Masson staining of the aortic sinus sections performed previously revealed markedly decreased collagen content in the plaques under *F. nucleatum* infection (Figure 1G). Accordingly, The expression of matrix metalloproteinase MMP2, MMP8, and MMP9 was significantly induced in infected macrophages or foam cells at 24 and 48 h (Figure 7D). FN is the most widely distributed and best characterized of all extracellular matrix proteins. Additionally, FN is demonstrated to be involved in regulating the physiologic activity of certain inflammatory cells, macrophage clearance of bacterial or non-bacterial particles, and extracellular matrix reorganization (Saba and Jaffe, 1980; Beezhold and Lause, 1987). It also contributes to the formation of atheromatous plaque (fibrous cap) and increases plaque stability (Chistiakov et al., 2013). Consistent with the *in vivo* data, *F. nucleatum* infection suppressed the FN1 expression and the secretion in macrophages and foam cells at 24 and 48 h (*p* < 0.05) (Figure 7E).

These *in vitro* data further demonstrate the pro-atherogenic effect of *F. nucleatum* on macrophages in a plaque microenvironment by promoting M1 polarization, releasing inflammatory mediators and matrix metalloproteinases, and through extracellular matrix degradation, which finally increases the risk of formation of vulnerable plaques.

## Discussion

The role of *F. nucleatum* in oral diseases and associated systemic effects are debatable. Previous studies support the hypothesis that *F. nucleatum* plays an intermediate bridging role in the pathogenesis of periodontal disease and atherosclerosis, mainly through assisting other periodontal bacteria by enhancing adhesion and invasion to host cells (Chukkapalli et al., 2015, 2017; Li et al., 2015). The virulence mechanisms of *F. nucleatum* include colonization and dissemination; the best-characterized and unique key virulence factor FadA adhesin/invasion binds to host cells, thus helping *F. nucleatum* to enter host cells independently, whereas other periodontal pathogens require additional factors, such as compromised mucosal integrity or coinfection with other microbes for passive invasion (Han et al., 2005; Saito et al., 2008; Han, 2015; Lee et al., 2019). FadA binds to cadherins, one of the cell-junction molecules, loosens cell–cell junctions, and further binds to VE-cadherin on endothelial cells to increase the permeability of the endothelial layer (Fardini et al., 2011). This may be one of the mechanisms of how *F. nucleatum* promotes the development of atherosclerosis. Meanwhile, it follows that *F. nucleatum* may act as an “enabling factor” to create conditions for the systematic dissemination of other periodontal pathogens. Moreover, *F. nucleatum* could induce host responses to stimulate factors predisposing to atherosclerosis, such as GroEL due to the cross-reactivity of GroEL antibodies with human heat shock protein 60 (Ford et al., 2005; Lee et al., 2012).

We found that *F. nucleatum* ATCC 25586 could invade vascular tissue and aggravate atherosclerotic lesions *via* periodontal infection. We also detected a higher abundance of *F. nucleatum* DNA copies in the aortic tissue in the infected group compared with the control group. Sequencing analysis showed that the *F. nucleatum* detected in the aorta was the strain *F. nucleatum* ATCC 25586 that was used for oral inoculation, indicating that *F. nucleatum* could migrate from the periodontium to aortic tissues. Invasion by periodontal pathogens is proposed as a possible mechanism of pathogenesis in periodontal and cardiovascular diseases. Previous studies report the presence of *F. nucleatum* DNA in clinical atherosclerotic tissue samples (Zaremba et al., 2007; Zhang et al., 2008; Figuero et al., 2011). Moreover, a higher *F. nucleatum* load was observed in atherosclerotic plaques with periodontitis than in plaques without periodontitis (Zhong et al., 2008). Next, *in vitro* experiments indicate the ability of *F. nucleatum* to highly invade the cytoplasm of macrophages as an obligate anaerobe. Confocal microscopy shows that this organism could maintain spindle-shaped rods after invading the cytoplasm, and a similar shape was also observed in Casasanta’s study (Xue et al., 2018; Casasanta et al., 2020). Previous studies report that *F. nucleatum* can survive for 12–72 h after the invasion in host cells, which provides the bacteria sufficient time to escape from the host defense mechanism and facilitate pathogenicity (Ji et al., 2010; Xue et al., 2018). Despite Velsko’s findings on the inherent atheroprotective effect of *F. nucleatum* (Velsko et al., 2015), we believe that the inconsistent results are due to different subspecies selected in the animal model studies. Different subspecies may have different pathogenicity, invasiveness, and the potential for systemic dissemination, resulting in different levels of disease activity (Huggan and Murdoch, 2008; Manson McGuire et al., 2014). In consideration of the heterogeneity among isolates, *Fusobacterium nucleatum* is divided into three subspecies that inhabit the human oral cavity by Dzink et al. (1990): *Fusobacterium nucleatum* subsp. *nucleatum* (*FNN*) with type strain ATCC 25586, which is the bacterium used in our study; *Fusobacterium nucleatum* subsp. *polymorphum*; and *Fusobacterium nucleatum* subsp. *vincentii* (*FNV*) with type strain ATCC 49256 as was used in Velsko’s study. *FNN* owns the highest numbers in plaque biofilm, whereas the least favorably adapted subspecies in the biofilm appears to be *FNV*, tightly involved in their pathogenic potency (Thurnheer et al., 2019). It is reported that the effect of *FNN* to reduce oxidative killing by neutrophils is stronger than that of *FNV*, which was suggested to mediate generalized paralysis of the immune system (Kurgan et al., 2017). *FNN* is also reported to be detected in the placentas of 80% of mice and the only subspecies to induce fetal miscarriage, but no evidence indicated that *FNV* could migrate and infect the placenta, suggesting that *FNN* is more prone to systemic infection than is *FNV* (Stockham et al., 2015).

A distinguishing feature of advanced atherosclerosis is the progressive accumulation of foam cells in plaques. Our study showed increased macrophages and deposition of lipid droplets in the artery wall of the infected group. The *in vitro* data showed that *F. nucleatum* could activate phagocytosis, enhance lipid accumulation, and increase CE levels in macrophages, further driving foam cell formation, which may also contribute to the increased apoptosis observed *in vivo* and the overall increase in plaque formation. This result was possibly caused by *F. nucleatum*-mediated modulation of cholesterol metabolism-related genes. We found that the expression of CD36, SR-A1, and LOX-1, the genes that mediate the internalization of ox-LDL, was upregulated during *F. nucleatum* stimulation and was further increased after co-culture with ox-LDL. SR-A1 was highly and significantly expressed in aortic tissues. Furthermore, *F. nucleatum* markedly increased the expression of ACAT-1 during foam cell formation. Conversely, we found that *F. nucleatum* decreased the levels of cholesterol transporters, such as ABCA1, ABCG1, and SR-BI in ox-LDL treated macrophages, which impaired the cellular cholesterol efflux pathway. These results could explain how *F. nucleatum* exacerbated lipid accumulation during foam cell formation. Our data are consistent with a previous study that used another periodontal pathogen and reported that *P. gingivalis* -LPS accelerated CE formation in macrophages by increasing ACAT-1 expression and reducing ABCG1 expression (Liu et al., 2016). Similarly, another study reported that *P. gingivalis* LPS exacerbated the formation of foam cells by increasing CD36 levels and decreasing ABCA1 levels in macrophages (Li et al., 2013). Our finding elucidated the mechanism of abnormal cholesterol metabolism induced by *F. nucleatum*.

Most studies suggest that M1 phenotype macrophages are more likely to lead to an acute atherothrombotic vascular event, whereas alternatively activated macrophages (M2), which are related to an anti-inflammatory expression pattern, play a preventive role in the progression of atherogenesis (Yang et al., 2020). There is no consensus on the effect of *F. nucleatum* on macrophage polarization. Xu et al. (2021) found that *F. nucleatum* participates in the reprograming of the tumor microenvironment by inducing M2 macrophage polarization, whereas M1 markers were not evaluated (Xu et al., 2021). However, some studies have contrary results. Wu et al. (2019) suggest that *F. nucleatum* AI-2, a major signal-type molecule that is involved in mediating communication among interspecies, induced macrophage M1 polarization by activating the TNFSF9/IL-1β pathway. Xue et al. (2018) found that *F. nucleatum* infection induces classical activation (M1-polarized) of THP-1-derived macrophages with the expression of the macrophage M1 markers CCR7, CXCR4, and HLA-DR increased and M2-polarized markers CD206 and CD163 reduced after live Fn or heated-kill Fn treated. In colitis, *F. nucleatum* can promote the progression of ulcerative colitis (UC) *via* proinflammatory M1 macrophage skewing *in vivo*, distinct from what is observed in a tumor setting; *F. nucleatum* alone did not alter expression of M1 or M2 markers obviously, but the common treatment of *F. nucleatum* and IFN-γ synergistically, which was used to simulate the inflammatory microenvironment, reduced M2 markers when compared to IFN-γ alone treatment (Liu et al., 2019). Similarly, our study found the increased proportion of M1-polarized macrophage in the atherosclerotic plaque microenvironment of an animal model after *F. nucleatum* infection (Figure 2B). *In vitro*, we co-cultured macrophage with ox-LDL to mimic foam cell formation during atherosclerosis and found that the M1 marker INOS upregulated and the M2 marker CD163 downregulated when macrophage was treated with *F. nucleatum* and ox-LDL synergistically, compared with ox-LDL alone treatment (Figure 7A). However, *F. nucleatum* alone had no significant effect on macrophage polarization. We believe that the immune response of macrophages to the microbes can be shaped based on the microenvironment (Martinez et al., 2009). Of note, our study is the first to evaluate the effect of *F. nucleatum* on the phenotypic transformation of macrophages and foam cells during atherosclerosis.

We observed that *F. nucleatum* reduced serum HDL-c levels and increased ox-LDL levels. Besides this, no significant difference was detected in TC, TG, or LDL-c levels between the two groups. HDL-c is an anti-atherosclerotic lipoprotein that reverses cholesterol transport, inhibits the oxidation of LDL, and neutralizes the atherogenic effects of ox-LDL (Parthasarathy et al., 1990). Ox-LDL, a key risk factor for atherosclerosis, is derived from LDL through modification by enzymes, such as phospholipases, and it contributes to the formation and progression of atherosclerotic plaques (Frostegård, 2013). Thus, in our study, the decreased serum HDL levels and increased serum ox-LDL levels in the infected group may contribute to disease development. A previous study also showed that ox-LDL levels were markedly enhanced, but no other lipid levels were altered during *P. gingivalis*-induced atherosclerosis (Velsko et al., 2014). Another study suggests a significant decrease in HDL-c levels by long-term *P. gingivalis* infection (Maekawa et al., 2011). The levels of inflammatory factors, such as TNF-α, IL-1β, IL-6, and the chemokine MCP-1 were considerably increased in serum and aortic tissues of the infected group. This is similar to *P. gingivalis* for which a study reports that *P. gingivalis* could induce the production of serum proinflammatory cytokines in Apoe^–/–^ mice (Pan et al., 2014). Our *in vitro* data also show that *F. nucleatum* promotes the secretion of inflammatory factors, such as TNF-α, IL-1β, IL-6, and the chemokine MCP-1 during foam cell formation, which suggests that the increased systemic inflammation level induced by *F. nucleatum* might accelerate atherosclerosis.

The levels of miR-155, miR-146a, and miR-23b in monocyte and macrophage could be induced after stimulated by LPS and ox-LDL, which are highly involved in regulating inflammatory responses (Li et al., 2016; He et al., 2018; Shao et al., 2018). Many studies report that miR-146a, miR-155, and miR-23b are important mediators that participate in the pathogenesis of atherosclerosis (Nazari-Jahantigh et al., 2012; Du et al., 2014; Bastami et al., 2016; He et al., 2018; Quan et al., 2018; Yin et al., 2019; Barraclough et al., 2020). In our study, miR−146a, miR-155, and miR-23b exhibited increased levels in the serum and aortic tissues of infected mice even though miR-155 expression in the aorta did not show a statistically significant increase. In addition, correlation analysis showed a positive relationship between serum miR−146a, miR-155, and miR-23b levels and plaque area and serum CRP levels. Thus, our studies reveal a preliminary association of miR-146a, miR-155, and miR-23b expression with *F. nucleatum*, suggesting that these microRNAs may directly or indirectly modulate or alter the process of atherosclerosis induced by *F. nucleatum*. To the best of our knowledge, this is the first study to report the association between *F. nucleatum* and atherosclerosis based on miRNA level analysis.

*F. nucleatum* is reported to trigger the production of matrix metalloproteinases by the host, which finally led to irreversible periodontal disease (Gursoy et al., 2010). Similarly in the atherosclerotic plaque microenvironment, the M1-polarized macrophages induced by *F. nucleatum* could secrete matrix metalloproteinase to cause plaque destabilization. It can be seen that the collagen fiber content in the plaques of *F. nucleatum*-infected mice decreased. We further found that the levels of MMP-2 and MMP-8 in aortic tissue significantly increased despite that the MMP-9 level was slightly increased with no significant difference in the *F. nucleatum* infected group compared with that in the control group. Consistently, our *in vitro* study showed that MMP-2, MMP-8, and MMP-9 levels were significantly increased under the stimulation of ox-LDL and *F. nucleatum* compared with those under ox-LDL treatment alone. Conversely, *F. nucleatum* inhibited the expression and secretion of extracellular matrix component FN1 *in vivo* and *in vitro*. A study suggests that the activation of macrophages by bacterial LPS could suppress fibronectin production, which helps inflammatory macrophages have better mobility to respond to the invading microorganisms (Trotter et al., 1989). However, the downregulation of FN1 may adversely affect the stable structure of the plaque. The low-circulating FN level could also increase the risk of coronary heart disease (Zhang et al., 2006).

To conclude, this study indicates the potential pathogenic effect of *F. nucleatum* on atherosclerosis. The results indicate a combination of distinct mechanisms as follows: (i) high invasiveness and an activated innate immune response, (ii) dyslipidemia, (iii) worsened systemic inflammatory burden, (iv) the induction of M1 macrophage skewing, foam cell formation and cell apoptosis, and (v) accelerated extracellular matrix degradation induced by matrix metalloproteinases, and all accelerate plaque formation and rupture. These findings provide insights into the role of *F. nucleatum* in the pathogenesis of atherosclerosis and that *F. nucleatum* might be considered a risk factor for cardiovascular disease.

## Data Availability Statement

The original contributions presented in the study are included in the article/Supplementary Material, further inquiries can be directed to the corresponding author/s.

## Ethics Statement

The animal study was reviewed and approved by the Ethics Committee of Sichuan University (NO. WCHSIRB-D-20 17-071).

## Author Contributions

JZ, LZ, and YW: conception and design, drafting the manuscript. All authors: acquisition, analysis, and interpretation.

## Conflict of Interest

The authors declare that the research was conducted in the absence of any commercial or financial relationships that could be construed as a potential conflict of interest.

## Publisher’s Note

All claims expressed in this article are solely those of the authors and do not necessarily represent those of their affiliated organizations, or those of the publisher, the editors and the reviewers. Any product that may be evaluated in this article, or claim that may be made by its manufacturer, is not guaranteed or endorsed by the publisher.

## Abbreviations

AS: Atherosclerosis
GroEL: Heat shock protein
ApoE^–/–^: Apolipoprotein E knockout
*F. nucleatum, Fn*: *Fusobacterium nucleatum*
ABR: Alveolar bone resorption
HP-1: The human monocytic cell line
dTHP-1: THP-1derived macrophages
MOI: Multiplicity of infection
CRP: C-reactive protein
MCP-1: Monocyte chemoattractant protein 1
MMP: Matrix metalloproteinase
FN1: Fibronectin 1
TC: Total cholesterol
TG: Total triglyceride
CE: Cholesteryl ester
HDL: High-density lipoprotein
LDL: Low-density lipoprotein
ox-LDL: Oxidized low-density lipoprotein
CD36: Cluster Determinant 36
SR-A1: Scavenger receptor A1
LOX-1: lectin-like ox-LDL receptor 1
ACAT1: cholesterol acyltransferase 1
ABCA1: ATP-binding cassette transporter A1
ABCG1: ATP-binding cassette transporter G1
SR-B1: Scavenger receptor B1.
