# Supplementary material for: Fusobacterium nucleatum Accelerates Atherosclerosis via Macrophage-Driven Aberrant Proinflammatory Response and Lipid Metabolism
Source: Front Microbiol. 2022 Mar 11;13:798685. doi: 10.3389/fmicb.2022.798685 (PMC8963492; doi:10.3389/fmicb.2022.798685)
Supplement: Supplementary file 1 [file Table_1.DOCX]

**Major Resources Table**

**Animals (in vivo studies)**

| **Species** | **Vendor or Source** | **Background Strain** | **Sex** | **Persistent ID / URL** |
| --- | --- | --- | --- | --- |
| ApoE^-/-^ mice | Beijing Vital River Laboratory Animal Technology Company | C57 | male | <https://www.vitalriver.com/> |

**Antibodies**

| **Target antigen** | **Vendor or Source** | **Catalog #** | **Working concentration** | **Persistent ID / URL** |
| --- | --- | --- | --- | --- |
| CD68 | Santa Cruz, California, USA | sc-20060 | 1:100 dilution | https://www.scbt.com/p/cd68-antibody-kp1?requestFrom=search |
| ABCA1 | Novus, USA | NB400-105 | 1:200 dilution | https://www.novusbio.com/products/abca1-antibody_nb400-105 |
| ABCG1 | Novus, USA | NB400-132 | 1:200 dilution | https://www.novusbio.com/products/abcg1-antibody_nb400-132 |
| INOS | Santa cruz, USA | sc-7271 | 1:200 dilution | https://www.scbt.com/p/nos2-antibody-c-11?requestFrom=search |
| CD163 | Abcam, USA | ab182422 | 1:200 dilution | https://www.abcam.cn/cd163-antibody-epr19518-ab182422.html |
| TLR2 | Affinity Biosciences, USA | DF7002 | 1:100 dilution | http://www.affbiotech.cn/goods-5804-DF7002-TLR2+Antibody.html |
| TLR4 | Affinity Biosciences, USA | AF7017 | 1:100 dilution | http://www.affbiotech.cn/goods-2070-AF7017-TLR4+Antibody.html |
| Caspase3 | Proteintech, USA | 19677-1-AP | 1:100 dilution | https://www.ptgcn.com/products/CASP3-Antibody-19677-1-AP.htm |
| Bax | abways, China | AY0553 | 1:100 dilution | http://abways.com/showproduct.asp?cid=AY0553 |
| IL-6 | Servicebio, China | GB11117 | 1:500 | <https://www.servicebio.cn/goodsdetail?id=1393> |
| IL-1β | Servicebio, China | GB11113 | 1:800 | <https://www.servicebio.cn/goodsdetail?id=1391> |
| TNF-α | Servicebio, China | GB11188 | 1:500 | <https://www.servicebio.cn/goodsdetail?id=4760> |
| IL-10 | Servicebio, China | GB11108 | 1:600 | <https://www.servicebio.cn/goodsdetail?id=1388> |
| MCP-1 | Servicebio, China | GB11199 | 1:1000 | <https://www.servicebio.cn/goodsdetail?id=1441> |
| MMP2 | Servicebio, China | GB11130 | 1:1000 | <https://www.servicebio.cn/goodsdetail?id=1399> |
| MMP8 | ImmunoWay Biotechnology, USA | YT2800 | 1:200 | <http://www.immunoway.com/Home/22/YT2800> |
| MMP9 | Servicebio, China | GB11132 | 1:1000 | <https://www.servicebio.cn/goodsdetail?id=1401> |

**Cultured Cells**

| **Name** | **Vendor or Source** | **Persistent ID / URL** |
| --- | --- | --- |
| Human monocyte cell line THP-1 (TIB-202) cells | American Type Culture Collection (ATCC) | <https://www.atcc.org/products/tib-202> |
| *F. nucleatum* ATCC25586 | American Type Culture Collection (ATCC) | <https://www.atcc.org/products/25586> |

**Other**

| **Description** | **Source / Repository** | **Persistent ID / URL** |
| --- | --- | --- |
| Brain heart infusion broth | BD Biosciences, CA, USA |  |
| microplate reader | Thermo Scientific, USA |  |
| tribromoethanol | Macklin, Shanghai |  |
| micro-CT scanner | SCANCO Medical AG, Fabrikweg2, CH-8306 Bruettisellen, Switzerland |  |
| TIANamp Genomic DNA Kit | DP304, Tiangen, China | <https://www.tiangen.com/?productShow/t1/1/id/26.html> |
| TB Green® Kit | Takara, Japan | [https://www.takarabiomed.com.cn/ProductShow.aspx? m=20141215102926123157&productID=20141227082641313454](https://www.takarabiomed.com.cn/ProductShow.aspx?m=20141215102926123157&productID=20141227082641313454) |
| qPCR System LightCycler® 480 II | Roche Diagnostics, Mannheim, Germany |  |
| CY3 Conjugated AffiniPure Goat Anti-mouse IgG | BA1031, Boster, China | <http://www.boster.com.cn/product/cy3-conjugated-affinipure-goat-anti-mouse-igg-h-l_ba1031.html> |
| DyLight 488 Conjugated AffiniPure Goat Anti-mouse IgG | BA1126, Boster, China | <http://www.boster.com.cn/product/dylight-488-conjugated-affinipure-goat-anti-mouse-igg-h-l_ba1126.html> |
| TUNEL kit | KGA7061, KEYGEN, China | <http://www.keygentec.com.cn/pro_detail.php?cid=102893&bid=435> |
| Goat Anti-Mouse IgG H&L (TRITC) antibody | HA1017, HUABIO, China | <http://www.huabio.cn/search?sort=asc&keyword=HA1017> |
| Goat anti-Rabbit IgG-FITC antibody | HA1004, HUABIO, China | <http://www.huabio.cn/product/Goat-anti-Rabbit-IgG-FITC-antibody-HA1004> |
| Goat anti-Rabbit IgG-AlexaFluor 488 | abs20025, Absin, China | <https://www.absin.cn/goat-rabbit-igg-alexafluor-488/abs20025.html> |
| Goat anti rabbit specific HRP conjugate | K5007, DAKO, USA |  |
| DAB | K5007, DAKO, USA |  |
| mouse serum oxidized low-density lipoprotein ELISA kit | CSB-E07933m, Cusabio, China | <http://www.cusabio.cn/ELISA-Kit/Mouse-oxidized-low-density-lipoprotein-OxLDL-ELISA-Kit-94762.html> |
| Mouse IL-6 ELISA Kit | 70-EK206/3-96, Lianke, China | <http://www.liankebio.com/product-598532.html> |
| Mouse TNF-α High Sensitivity ELISA Kit | 70-EK282HS-96, Lianke, China | <http://www.liankebio.com/product-598580.html> |
| Mouse IL-1 beta ELISA Kit | EK0394, Boster, China | <http://www.boster.com.cn/product/human-il-1-beta-elisa-kit_ek0392.html> |
| C Reactive Protein (PTX1) Mouse ELISA kit | ab157712, abcam, USA | <https://www.abcam.cn/mouse-c-reactive-protein-elisa-kit-ptx1-ab157712.html?#description_protocols> |
| Mini Samples ELISA Kit for Monocyte Chemotactic Protein 1 (MCP1) | MEA087Mu, Cloud-Clone Corp, China | <http://www.uscnk.cn/uscn/Mini-Samples-ELISA-Kit-for-Monocyte-Chemotactic-Protein-1-(MCP1)-MEA087Mu.htm> |
| miRNeasy Mini Kit | 217004, Qiagen, USA | <https://www.qiagen.com/us/products/discovery-and-translational-research/dna-rna-purification/rna-purification/mirna/mirneasy-kits/?catno=217004> |
| miRNA extraction Kit | DP503, TIANGEN, Beijing, China | <https://www.tiangen.com/?productShow/t1/3/id/375.html> |
| Mir-X miRNA First-Strand Synthesis Kit | Takara Bio, Japan | [https://www.takarabiomed.com.cn/ProductShow.aspx? m=20141220144728857981&productID=20141227114408830191](https://www.takarabiomed.com.cn/ProductShow.aspx?m=20141220144728857981&productID=20141227114408830191) |
| PrimeScript RT Reagent Kit | Takara Bio, Japan | [https://www.takarabiomed.com.cn/ProductShow.aspx? m=20141215102916640154&productID=20141224095630690325](https://www.takarabiomed.com.cn/ProductShow.aspx?%20m=20141215102916640154&productID=20141224095630690325) |
| exogenous Caenorhabditis elegans miRNA | CR100-01, Tiangen, China | <https://www.tiangen.com/?productShow/t1/3/id/359.html> |
| RPMI1640 medium | Gibco, USA |  |
| Fetal Bovine Serum | Gibco, USA |  |
| penicillin–streptomycin | Gibco, USA |  |
| phorbol 12-myristate 13-acetate | P8139, Sigma-Aldrich, USA | <https://www.sigmaaldrich.cn/CN/zh/product/sigma/p8139?context=product> |
| Annexin-V-PI Staining kit | KGA103, KeyGEN Bio TECH, Nanjing, China | <http://www.keygentec.com.cn/pro_detail.php?cid=102866&bid=446> |
| 2μm polystyrene fluorescent carboxylate-modified microspheres | Invitrogen, Carlsbad, CA | <https://www.thermofisher.cn/order/catalog/product/F8826?#/F8826> |
| Cholesterol Quantitation Kit | CS0005-1KT, Sigma, USA | <https://www.sigmaaldrich.cn/CN/zh/product/sigma/cs0005?context=product> |
| TRIzol reagent | Takara Bio, Japan |  |
| Human IL-6 ELISA Kit | EK106/2-96, Lianke, China | <http://www.liankebio.com/product-598040.html> |
| Human IL-1β ELISA Kit | EK101B-96, Lianke, China | <http://www.liankebio.com/product-597960.html> |
| Human TNF-a ELISA Kit | EK182-96, Lianke, China | <http://www.liankebio.com/product-598240.html> |
| Human MCP-1/CCL2 ELISA Kit | EK187-96, Lianke, China | <http://www.liankebio.com/product-597644.html> |
| Human Fibronectin, FN ELISA Kit | CSB-E04551h, Cusabio, China | <http://www.cusabio.cn/ELISA-Kit/Human-Fibronectin-FN-ELISA-Kit-78906.html> |
