# Supplementary material for: Fusobacterium nucleatum Accelerates Atherosclerosis via Macrophage-Driven Aberrant Proinflammatory Response and Lipid Metabolism
Source: Front Microbiol. 2022 Mar 11;13:798685. doi: 10.3389/fmicb.2022.798685 (PMC8963492; doi:10.3389/fmicb.2022.798685)
Supplement: Supplementary file 2 [file Data_Sheet_1.docx]

Supplementary Material

# Supplementary Data

**SUPPLEMENTARY METHODS**

**1.1 In vivo study**

**Bacterial strains, Animals, and Oral Infection model**

*F. nucleatum* ATCC 25586 was grown anaerobically on blood agar plates (BD Biosciences, CA, USA) at 37℃ for 2–6 days and then incubated in the brain heart infusion broth (BD Biosciences, CA, USA) for 2 days. *F. nucleatum* cultured in the logarithmic growth phase was harvested by centrifugation at 4,000 rpm for 10 min and washed three times with phosphate buffered saline (PBS, HyClone, USA). The bacterial concentration was spectrophotometrically standardized to optical density (OD) value at 540 nm = 0.8 *F. nucleatum* by using a microplate reader (Thermo Scientific, USA), corresponding to 1×10^9^ bacteria/ml [1]. Then the bacterial suspensions were centrifuged and diluted by 4% Carboxymethyl Cellulose (CMC)-PBS (Sigma, USA), reaching the concentration of 1×10^9^ bacteria/ml. Six-week-old male ApoE^-/-^ mice (Beijing Vital River Laboratory Animal Technology Company) were housed in a specific pathogen-free controlled animal laboratory under 12-h dark / 12-h light photophase and fed with a standard chow diet [2, 3]. Before oral inoculation, all mice (n= 16, in total) were administered with sulfamethoxazole and trimethoprim daily in the drinking water, and concurrently the oral cavities were rinsed with 0.12% chlorhexidine gluconate for 10 days to inhibit the colonization of other microorganisms, followed by a 3-day antibiotic wash-out period [4]. Then, the mice were randomly assigned to 2 groups: the infected group received oral inoculation with 100 µL *F. nucleatum* suspended in 4%CMC-PBS (1×10^9^ bacteria/ml), while the sham-infected group received the same volume of sterile vehicle (4%CMC-PBS) (n = 8, each group)[5-7]. The oral inoculation was performed every alternate day for 12 weeks. The body weight of each mouse was recorded weekly during the experimental period.

All mice were sacrificed via exsanguination under anesthesia with 1.2% tribromoethanol (Avertin; 240 mg/Kg; intraperitoneal injection) at the 15^th^ week of the experiment [8]. The blood, maxilla and mandibles, gingival, aorta， and hearts of the animals were harvested. The serum was separated and stored at -80℃ from the blood for cholesterol measurement, qRT-PCR and ELISA. The soft tissues from the left maxillae and mandibular were removed for bone resorption measurement. The right maxillae and mandibles, the aorta and the hearts were immediately fixed in 10% neutral buffered formalin for [histologic](javascript:;)al [section](javascript:;)ing. The remaining aortic tissues were stored at -80℃. All animal procedures performed conformed to the guidelines from the Directive 2010/63/EU of the European Parliament on the protection of animals used for scientific purposes and approved by the Ethics Committee of Sichuan University (NO. WCHSIRB-D-2017-071).

**Alveolar bone resorption analysis**

The left maxillae and mandibles were scanned by using a high-resolution micro-CT scanner (SCANCO Medical AG, Fabrikweg2, CH-8306 Bruettisellen, Switzerland). Data were acquired at 70 kVp, with a 10-µm isotropic voxel size. Then, all data were collected and reconstructed, visualized, and analyzed by using SCANVO Medical Visualizer software. The horizontal alveolar bone resorption (ABR) area was measured referring to previous studies[9-11]. The area between the cemento-enamel junction (CEJ) and the alveolar bone crest (ABC) of molars 1, 2, and 3 of maxilla palatal, maxilla buccal, and mandible lingual sides was measured in mm^2^ using the ImageJ software. Then, we sum them up to calculate the total ABR and compare it between the two groups.

**Absolute qPCR of bacterial genomic DNA in aortic tissues**

To evaluate the colonization of *F. nucleatum* through hematogenous dissemination in the aortic tissue, we homogenized aortic tissues and extracted the genomic DNA by using the TIANamp Genomic DNA Kit (DP304; Tiangen, China) as per the manufacturer’s protocol. The isolated DNA was suspended in 50-µL of the TE buffer and quantified by an Agilent 2100 bioanalyzer (Agilent Technologies, Santa Clara, CA, USA). The standard curve (Supplementary Figure 3) was constructed using cycle threshold (CT) values of 10-fold serial dilution (from 10^7^ to 10^1^ copies ul^-1^) of the DNA products purified after PCR amplification (template: *F. nucleatum* DNA) and its copies. The copies of PCR products were calculated as follows: DNA (copies/µL) = 6.02 × 10^23^ (copies/mol) × DNA concentration (ng/µL) × 10^–9^ (g/ng) /DNA length (bp)/660 (g/mol/bp) [12].

The quantitative polymerase chain reaction (qPCR) was performed by using the TB Green® Kit (Takara, Japan) on the qPCR System LightCycler® 480 II (Roche Diagnostics, Mannheim, Germany) according to the manufacturer’s instructions. The primers of *F. nucleatum* ATCC25586 16S rDNA (designed by TSINGKE Biological Technology): 5’-GGCCACAAGGGGACTGAGACA-3’ (forward) and 5’- TTTAGCCGTCACTTCTTCTGTTGG-3’ (reverse). Amplification reactions were conducted as follows: initial denaturation at 95oC for 30 sec, denaturation at 95oC for 5 sec, annealing/extension at 60oC for 30 sec, and 40 cycles for amplification. The corresponding copy number was calculated using the standard curve. Then, the qRT-PCR products were sent to the TSINGKE Biological Technology company for sequencing. The sequencing results were analyzed by the BLAST nucleotide search engine to perform species and subspecies identification with reference to the full-length 16S rRNA gene sequences of the standard sequence in the NCBI GeneBank.

**En face morphometric analysis of the aortic tree**

Oil Red O staining was performed to assess the atherosclerotic lesions in the aortic tree. Opened aortas longitudinally, briefly rinsed with 78% methanol, and stained with 0.16% Oil Red O solution for 50 min was then placed in 78% methanol for 5 min, followed by imaging with a stereomicroscope. The lesion area was quantified as the Oil Red O staining positive area, and their percentage in the total aorta area was determined via the ImageJ software [13].

**Histology, immunohistochemistry, immunofluorescence, and morphometric analyses**

The right maxillae and mandibles were fixed in 10% neutral buffered formalin for 24 h. The tissue was decalcified, embedded, sectioned, and finally stained with H&E and Trap [14]. The degree of inflammation, the type of inflammatory cells, osteoclast, apical migration of the junctional epithelium, and epithelial hyperplasia was recorded [15]. The cytokines IL-6, IL-1β, IL-17 and TNF-α in gingiva tissue was further evaluated by immunohistochemistry. The tissue sections were deparaffinized and hydrated. Then, they were incubated in 0.1% hydrogen peroxide in distilled water for blocking the activity of endogenous peroxidase and interspersed by washing with PBS. Then, they were incubated with specific primary antibodies for IL-6 (1:500, GB11117; Servicebio, China), IL-1β (1:800, GB11113; Servicebio, China), IL-17(1:500, GB11110; Servicebio, China) and TNF-α (1:500, GB11188; Servicebio, China) overnight at 4℃, followed by washed and incubated with secondary goat anti-rabbit-specific HRP conjugate (K5007; Dako, USA) for 1 h. The sections were rinsed and developed with DAB (K5007; Dako, USA), and the nuclei were stained with hematoxylin[16]. The interproximal areas between the molars in each section were pictured with a microscope (Leica, Germany).

Some of the mouse hearts were fixed in 10% neutral buffered formalin and embedded in paraffin, while others were submersed in 4% paraformaldehyde (PFA) overnight, followed by transferring to 30% sucrose until the next day. Subsequently, the hearts were embedded in OCT and frozen, and 5-µm-thick sections were cut. Every fifth slide from the serial sections was stained with H&E or Oil Red O for quantification of the lesion area [13]. A total of 5 sections from the same mouse were obtained to calculate the average size of the aortic lesion areas. Morphometric analyses of the atherosclerotic plaque were performed as described previously, whereby the fibrous cap and necrotic core area were measured as the percentage of the total plaque area [17]. Necrotic core was defined as an H&E staining-free clear area [18]. Collagen content was assessed by Masson staining of the consecutive slides. To evaluate the macrophage content in the aortic sinus, immunofluorescence staining was performed with primary antibodies against CD68 (1:100, Santa Cruz, California, USA). DyLight 488 Conjugated AffiniPure Goat Anti-Mouse IgG (BA1126, Boster, China) and CY3 Conjugated AffiniPure Goat Anti-Mouse IgG (BA1031, Boster) were applied as the secondary antibodies. The primary antibodies against ABCA1(NB400-105, Novus), ABCG1(NB400-132, Novus), INOS (sc-7271, Santa cruz, USA) and CD163(ab182422, Abcam, USA), as well as the secondary antibody Goat Anti-Mouse IgG H&L (TRITC) antibody (HA1017, HUABIO, China), Goat anti-Rabbit IgG-FITC antibody (HA1004, HUABIO, China), and Goat anti-Rabbit IgG-AlexaFluor 488 (abs20025; Absin, China) for immunofluorescence staining. The cell nucleus was counterstained with DAPI for 10 min. The apoptotic cells in the lesions were stained with the TUNEL kit (KGA7061; Keygen, China). The apoptosis rate of macrophages was exhibited as the number of TUNEL-positive cells mm^-2^ CD68-positive area in the [atherosclerotic](javascript:;) [plaque](javascript:;)s [13]. The M1/M2 macrophage ratio was calculated from their positive areas [19]. ABCA1 and ABCG1 immunoreactivity was normalized to the lesion size [20]. The levels of TLR-2, TLR-4, Caspase 3 Bax, IL-6, IL-1β, TNF-α, IL-10, MCP-1, MMP2, MMP8, and MMP9 levels in the aortas were assessed by immunohistochemistry [2]. The [paraffin](javascript:;) [section](javascript:;)s of the aortic sinus were incubated with specific primary antibodies for TLR2,TLR4 (1:100, DF7002 and AF7017, [Affinity Biosciences](https://www.baidu.com/link?url=NoCciYYeBXEqB5-ZfVJckVRPFK3HFNQj_tN3s-AE1ntAmG6ja1V99fpYW9VCVRJ2&wd=&eqid=dfc73a9b00006ced000000065e476d42), USA), Caspase 3 (1:100, 19677-1-AP, Proteintech, USA), Bax (1:100, AY0553, Abways, China), IL-6 (1:500, GB11117; Servicebio), IL-1β (1:800, GB11113; Servicebio), TNF-α (1:500, GB11188; Servicebio), IL-10 (1:600, GB11108; Servicebio), MCP-1 (1:1000, GB11199; Servicebio), MMP2 (1:1000, GB11130; Servicebio), MMP8 (1:200, YT2800; ImmunoWay Biotechnology, USA), MMP9 (1:1000, GB11132, Servicebio) overnight at 4℃. Next, they were washed and incubated with secondary goat anti-rabbit-specific HRP conjugate (K5007; Dako, USA) for 1 h. The sections were rinsed and developed with DAB (K5007; Dako, USA), and the nuclei were stained with hematoxylin. Finally, all staining sections were captured by using a microscope (Leica) and subjected to quantifying analyses by the ImageJ software.

**Serum lipid and** **cytokine analysis**

Total cholesterol (TC), total triglycerides (TGs), low-density lipoprotein cholesterol (LDL-c), and high-density lipoprotein cholesterol (HDL-c) in the serum were detected by using an automatic biochemistry analyzer (Chemray 240; Rayto, China) [2]. The serum ox-LDL levels were determined using the mouse serum oxidized low-density lipoprotein ELISA kit (Cusabio, China) [21]. The concentrations of IL-6, TNF-α, IL-1β, CRP and MCP-1 in the serum were quantified by using immunoassay kits in accordance with the manufacturer’s protocols [22]. The following ELISA kits were used in the present study: IL-6 (Lianke, China), TNF-α (Lianke), IL-1β (Boster), CRP (Abcam), and MCP-1(Cloud-Clone Corp, China).

**Quantitative real-time PCR analysis of serum and aortic tissues**

For analyzing the expression of genes and miRNAs associated with atherosclerosis, total RNAs of the aorta samples and miRNAs of the serum were extracted by using the miRNeasy Mini Kit (Qiagen, USA) and the miRNA Extraction Kit (DP503; Tiangen) as per the respective manufacturer's instruction. RNA concentration and integrity were determined by using the Agilent 2100 Bioanalyzer (Agilent Technologies, Santa Clara, CA, USA). The Mir-X miRNA First-Strand Synthesis Kit (Takara Bio, Japan) was used for miRNA reverse-transcription, while the PrimeScript RT Reagent Kit (Takara Bio) was used for cDNA synthesis. qRT-PCR was performed in triplicates by using the TB Green® Kit (Takara Bio) on the QPCR System LightCycler® 480 II (Roche Diagnostics). The relative miRNA expression was normalized to the U6 expressionin aortic tissues and exogenous Caenorhabditis elegans miRNA (CR100-01, Tiangen) in the serum samples. The relative mRNA expression was normalized to those of GAPDH in the aortic tissues. Data were collected and quantitatively analyzed with the 2^−ΔΔ^*^ct^* method. Reverse primers of miRNAs and U6 primers were provided from the Mir-X miRNA First-Strand Synthesis Kit (Takara Bio). The remaining primer sequences are listed in **[Table S1](https://www.sciencedirect.com/science/article/pii/S0003996918306502?via%3Dihub#tbl0005)**.

**1.2 In vitro study**

**Cell culture and Co-culture model**

Human monocyte cell line THP-1 (TIB-202) cells were purchased from American Type Culture Collection (ATCC) and cultured in the RPMI1640 medium (Gibco, USA) supplemented with 10% FBS (Gibco, USA), 1% 100 U/mL penicillin–streptomycin (Gibco, USA) under 5% CO2 atmosphere at 37°C. Subsequently, THP-1 monocytes were differentiated into macrophages (THP-1- derived macrophages, dTHP1) after treatment with 100-nM PMA (Sigma-Aldrich) for 48 h. The antibiotics were removed from the medium before co-culturing the bacterial and host cells. *F. nucleatum* was centrifuged and suspended in the RPMI 1640 (Gibco, USA). After the concentration was measured by using a microplate reader [1], the bacterial suspensions were added into dTHP1 cells at different MOI (multiplicity of infection, bacteria: cells) of 0,10,100,200, and 500, followed by co-culturing to the indicated time points (24 h, 48 h) under a humidiﬁed 5 % CO2 atmosphere at 37°C. The morphology of dTHP1 cells was observed and captured by using a light microscope (Olympus, Japan).

**Flow cytometry analysis of apoptosis**

THP-1 cells were seeded at a cell density of 1 × 10^6^ cells mL^−1^ in a 6-well plate and differentiated into macrophages under 100-nM PMA for 48 h. Next, macrophages were treated with bacteria at different MOI (0,10,100,200, and 500) for the indicated time points (24 h and 48 h), as described earlier. Apoptosis was evaluated in 3 independent experiments using the Annexin-V-PI Staining Kit (KeyGEN Bio TECH, Nanjing, China). According to the kit’s instructions, the cells were collected and washed twice with PBS, followed by incubation with 100μL of the buﬀer solution, 5μL of Annexin V-FITC and 5μL of propidium iodide (PI) for 15 min in the dark at room temperature. The percentage of apoptotic cells was the sum of late apoptotic cells (Annexin V+/PI+) and the early apoptotic cells (Annexin V+/PI+). Flow cytometry was performed by using a FACS ﬂow cytometer (Beckman-Coulter, Miami, USA) and analyzed on the FlowJo software (TreeStar, Oregon, USA) [23].

**Immunofluorescence**

The invasion assay was performed as described earlier, and *F. nucleatum* infected dTHP-1 cells at an MOI of 100 for 24 h. The resultant cells were washed with PBS at least thrice to remove the non-adherent bacteria and then fixed with 4% PFA, followed by permeabilization with 0.2% Triton X-100. The macrophages were visualized with CD68 antibody (Santa Cruz, USA). DAPI was used to stain cell nuclei and label bacteria [24]. Multiple images were captured at different focal points (Z-stack) obtained for temporal-spatial visualization of bacteria under confocal laser scanning microscopy (Olympus, Japan).

**Phagocytosis assay**

The macrophage phagocytic activity was assayed using 2μm of polystyrene fluorescent carboxylate-modified microspheres (Invitrogen, Carlsbad, CA). Before starting the phagocytosis assay, dTHP-1 cells were co-cultured with *F. nucleatum* for 2 or 24 h at an MOI of 100, and the fluorescent microspheres were opsonized by mixing with 10% FBS-PBS for 1 h. Following opsonization, the fluorescent microspheres were suspended into normal culture media and added to the plates with the dTHP-1 cells and removal of the original medium at a ratio of 50 microspheres/plated cell, followed by incubation for 2 h [25]. Then, the cells were harvested, washed twice with cold flow buffer (2% BSA–0.01% NaN3-PBS), and resuspended in 200μL of flow buffer for flow cytometry analysis. The phagocytic capacity was expressed as the number of cells positive for fluorescent microspheres, while the phagocytic efficiency was determined by the M.F.I. of the positive cells [26]. All samples were performed in triplicate. To visualize the phagocytosis process, after incubation with fluorescent microspheres, the cells were co-incubated with CD68 antibody, and then stained with FITC and DAPI. The slides were collected and analyzed at room temperature by a confocal laser scanning microscopy (Olympus).

**Oil Red O staining and cellular cholesterol measurement**

Oil Red O staining was performed to observe lipid accumulation in macrophages. Then, 50 µg/ mL of ox-LDL was applied to induce foam cell formation derived from THP-1 macrophages [27]; meanwhile, the cells were treated with *F. nucleatum* at an MOI of 100 for 24 h and 48 h. For Oil Red O staining, the cells were fixed in 4% PFA and stained with Oil Red O [28]. After capturing the images by microscopy (Leica), the density of the lipid content was examined via alcohol extraction. The absorbance at 540 nm was evaluated by using a microplate reader (ThermoFisher, USA).

Intracellular TC and free cholesterol (FC) content were determined by using the Cholesterol Quantitation Kit (CS0005-1KT; Sigma, USA) according to the manufacturer’s instructions. The cholesteryl ester (CE) content was obtained from TC after subtracting the FC content [29].

**RNA isolation and quantitative real-time PCR**

The total RNA of the cells was extracted with the TRIzol reagent (Takara Bio). Total RNA (1 g) from each sample was used for cDNA synthesis using the PrimeScript RT Reagent Kit (Takara Bio). Quantitative RT-PCR was performed with the QPCR System LightCycler® 480 II (Roche Diagnostics) and the SYBR Premix Ex Taq II (Takara Bio. Japan) according to the manufacturer’s instructions. The relative levels were calculated using the 2^−ΔΔCt^ method after the normalization to GAPDH. The primers used in the qRT-PCR reaction are presented in **Table S2**.

**Cytokine analyses**

After infection with *F.* nucleatum for 24 h and 48 h [as](javascript:;) [mentioned](javascript:;) [above](javascript:;), the culture supernatants of the cells were collected to quantify the levels of cytokines IL-1β, [TNF-α](https://www.sciencedirect.com/topics/medicine-and-dentistry/tumor-necrosis-factor), IL-6 ,MCP-1, and FN1. ELISA kits (Lianke, China) were applied in this assay in accordance with the manufacturer’s protocols.

**1.3 Statistical** **analyses**

All statistical data were analyzed by using the SPSS version 21.0 software. Comparisons between the groups were performed using Student's t-test. For multiple comparisons, the data were analyzed by one-way analysis of variance (ANOVA) with post hoc Tukey. Spearman correlation analysis was conducted to assess the correlation between the extent of [atherosclerotic](javascript:;) lesion and the serum microRNA levels. The data were characterized as mean ± SD of at least 3independent experiments unless otherwise specified. P< 0.05 was considered to be statistically significant.

# Supplementary Figures and Tables

## Supplementary Figures


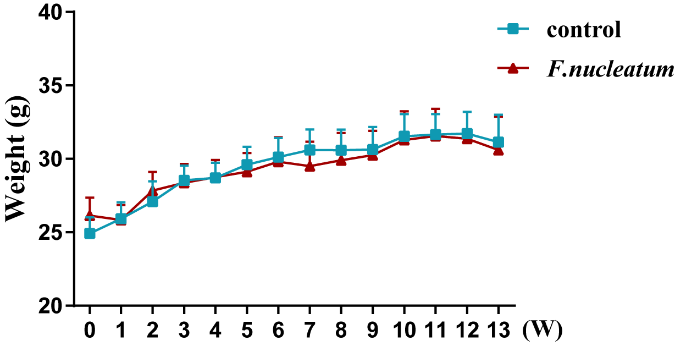


**Supplementary Figure 1.** Body weight of mice were measured weekly from infection baseline to euthanasia. (n=8, each group).


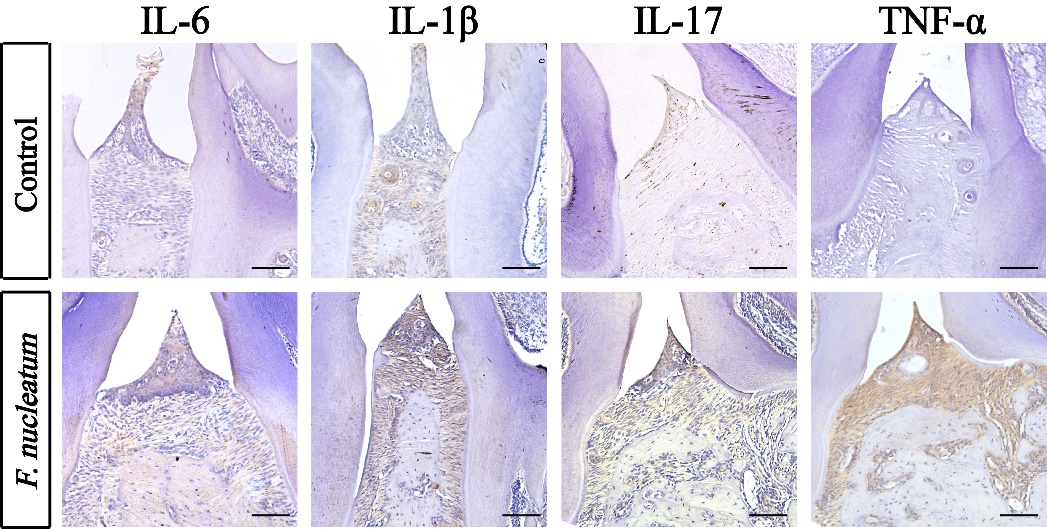


**Supplementary Figure 2.** The levels of cytokines IL-6, IL-1β, IL-17 and TNF-α in gingival were examined by immunohistochemistry. Scale bars, 100 μm.


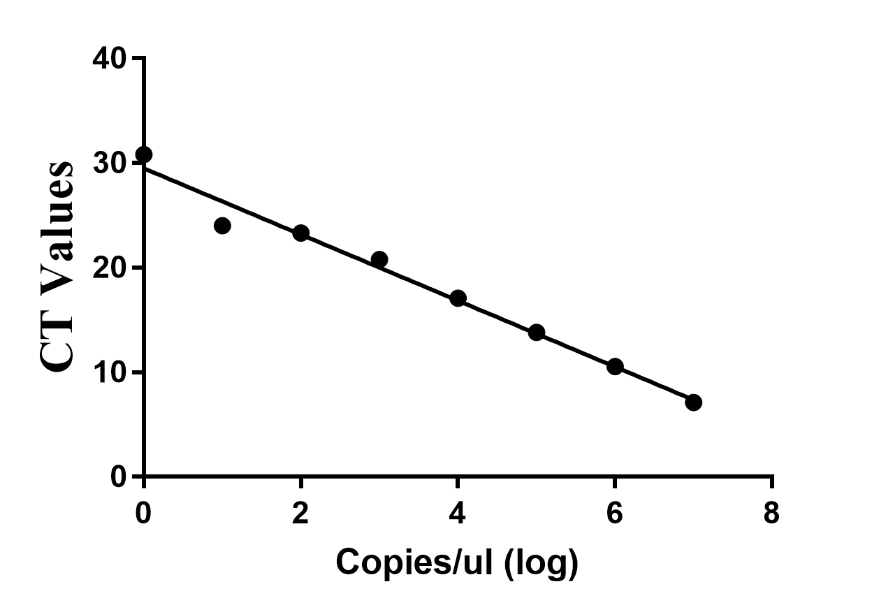


**Supplementary Figure 3.** **The standard curve of *F. nucleatum* DNA in absolute qPCR analysis.**

**
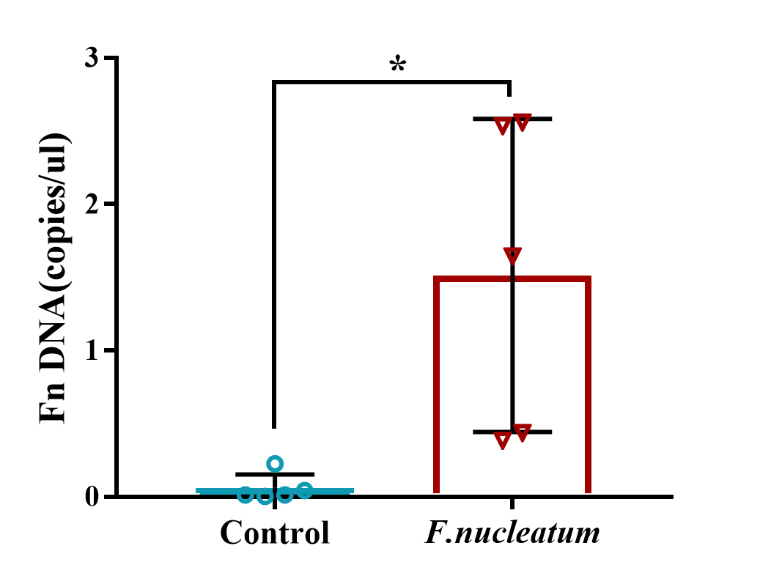
**

**Supplementary Figure 4.** Absolute qPCR analyzes the load of *F. nucleatum* genomic DNA in aortic tissues quantified as copies (n = 5, each group). Data represent the mean ± SD. *p < 0.05 vs control group. Student's t-test.

## Supplementary Tables

**Table S1.** Primer Sequences Used In vivo Study

| **Genes** | **Forward primer 5′ to 3′** | | **Reverse primer 5′ to 3′** |
| --- | --- | --- | --- |
| mGAPDH | GGTTGTCTCCTGCGACTTCA | | TGGTCCAGGGTTTCTTACTCC |
| mTLR2 | CTCCCAGATGCTTCGTTGTTCCC | | GTTGTCGCCTGCTTCCAGAGTC |
| mTLR4 | ACCTGGCTGGTTTACACGTC | | CTGCCAGAGACATTGCAGAA |
| mCASP3 | AGTGGGACTGATGAGGAGATGGC | | ATGCTGCAAAGGGACTGGATGAAC |
| mBAX | CGTGAGCGGCTGCTTGTCTG | | ATGGTGAGCGAGGCGGTGAG |
| mIL-6 | TAGCTACCTGGAGTACATGAAGAACA | | TGGTCCTTAGCCACTCCTTCTG |
| mIL-1β | CAACCAACAAGTGATATTCTCCATG | | ATCCACACTCTCCAGCTGCA |
| mTNF-α | CATCTTCTCAAAATTCGAGTGACAA | | TGGGAGTAGACAAGGTACAACCC |
| mIL-10 | TCCCTGGGTGAGAAGCTGAAGAC | | CACCTGCTCCACTGCCTTGC |
| mMCP-1 | CTCACCTGCTGCTACTCATTC | | CTTCTTTGGGACACCTGCT |
| mMMP-2 | GTGCCAAGGTGGAAATCAG | | GTTGAAGGAAACGAGCGAA |
| mMMP-8 | TGGGCTCTAAGTGGCTATGACCTG | | ATCAATGGCTTGGACACTCCTTGG |
| mMMP-9 | CTGGACAGCCAGACACTAAAG | | CTCGCGGCAAGTCTTCAGAG |
| mFN1 | AGTGGCTGAAGTCGCAAGGAAAC | | TAAGTCTGGGTCACGGCTGTCTC |
| mCD68 | CCTCTTGCTGCCTCTCATCATTGG | | GGCTGGTAGGTTGATTGTCGTCTG |
| mF4/80 | TTCCTGCTGTGTCGTGCTGTTC | | GCCGTCTGGTTGTCAGTCTTGTC |
| mCSF1 | CCAATGCTAACGCCACCGAGAG | | GCCTTGTTCTGCTCCTCATAGTCC |
| miNOS | ACTCAGCCAAGCCCTCACCTAC | | TCCAATCTCTGCCTATCCGTCTCG |
| mCD163 | AATCACATCATGGCACAGGTCACC | | TCGTCGCTTCAGAGTCCACAGG |
| mCD36 | GCAGGTCTATCTACGCTGTGTTCG | | TGTCTGGATTCTGGAGGGGTGATG |
| mSR-A1 | GCGGGAGGTCCTGTATGACT | | CGTCGAGACCCTTTCTCCCT |
| mLOX1 | TGAAGCCTGCGAATGACGAG | | GTCACTGACAACACCCAGGCAGAG |
| mACAT-1 | TCCACTCCATGCACCACAGTAAAC | | CGCCTGCCACCATCACATCC |
| mABCA1 | AGAAGGAGGCTCGGCTGAAGG | | GAGGGATGAGGCTGCTAACAAACC |
| mABCG1 | CATGCTGCTGCCTCACCTCAC | | TCTCGTCTGCCTTCATCCTTCTCC |
| mSR-B1 | GAGCAAGCCTGTGAGCCTGAAG | | GCATGTCTGGGAGGTACGTGTTG |
| mmu-miR-146a | | CGCGTGAGAACTTAATTCCATGGGTT | |
| mmu-miR-155 | | CGCCGTTAATGCTAATTGTGATAGGGG | |
| mmu-miR-23b | | CGCATCACATTTCCAGGGATTACCAC | |

**Table S2.** Primer Sequences Used In vitro Study

| **Genes** | **Forward primer 5′ to 3′** | **Reverse primer 5′ to 3′** |
| --- | --- | --- |
| hGAPDH | CTTTGGTATCGTGGAAGGACTC | GTAGAGGCAGGGATGATGTTCT |
| hCASP3 | TCACAGCAAAAGGAGCAGTT | TCACAGCAAAAGGAGCAGTT |
| hBAX | TCCACCAAGAAGCTGAGCGAG | GTCCAGCCCATGATGGTTCT |
| hCD36 | GGTGATGATGGAGAATAAGCC | AAGAGCCCAGAGTCGGAGTTG |
| hSR-A1 | GGACAGGGACAGAGATAGGGACAG | ACAGGAGGACGACGAGGATGAAG |
| hLOX-1 | CTTGCTCGGAAGCTGAATG | CCGTCCTCCCAGAGCCAT |
| hACAT-1 | CGGCAGATGCAGCGAAGAGG | AATAGGTTCTACAGCAGCGTCAGC |
| hABCA1 | GCTGGCCTGGATTTACTCAG | CCAGCTAAACCAGAGGATGC |
| hABCG1 | TTCGACCAGCTTTACGTCCT | ATCTGCTGGGTTGTGGTAGG |
| hSR-B1 | TTCCAGTTCCAGCCCTCCAA | GAGGGTGGTGAATGCCAAGG |
| hiNOS | AGCCTGAGAGACAGAGGCTG | CCATCTCGGGTGTGGTAGGT |
| hCD163 | GACGATGCTCAGGTGGTGTG | GGCAGGACAATCCCACAAGG |
| hIL-6 | CAGGAGAAGATTCCAAAGAT | CTCTTGTAACATGTCTCCTT |
| hIL-1β | GAACTGAAAGCTCTCCACCT | TCCCATCTTCTTCTTTGGGT |
| hTNF-α | GTAGCCCATGTTGTAGCAAA | CCTGGGAGTAGATGAGGTACA |
| hMCP-1 | CCCCAGTCACCTGCTGTT | CTGCTTGGGGTCAGCACA |
| hMMP-2 | TGATGGCATCGCTCAGATCC | GGCCTCGTATACCGCATCAA |
| hMMP-8 | CTTTCAGGGAAACCAGCAAC | GCTTGGTCCAGTAGGTTGGA |
| hMMP-9 | CCTGGGCAGATTCCAAACCT | GTACACGCGAGTGAAGGTGA |
| hFN1 | CAAGAAGGGCTCGTGTGACAGATG | TGGCTGGAACGGCATCAACTTG |

**Table S3.** The sequence results of qRT-PCR products in aortic tissues based on primers of *F. nucleatum* 16S rDNA.

|  | **Sequence** |
| --- | --- |
| **F-** | GATCGTGACTTCACTGAAGCACTTTACATTCCGAAAAACGTCATCGTGCACACAGAATTGCTGGATCAGACTCTCGGTCCATTGTCCAATATTCCCCACTGCTGCCTCCCGTAGGAGTAAGGGCCGTGTCTCAGTCCCCTTGTGGCCAAA |
| **R-** | CAAATTCACGGGAGGAGCAGTGGGGATATTGGACAATGGACCGAGAGTCTGATCCAGCAATTCTGTGTGCACGATGACGTTTTTCGGAATGTAAAGTGCTTTCAGTTGGGAAGAAAAAAATGACGGTACCAACAGAAGAAGTGACGGCTAAAAAAG |

**References**

1. Wang Q, Zhao L, Xu C, Zhou J, Wu Y. Fusobacterium nucleatum stimulates monocyte adhesion to and transmigration through endothelial cells. *Arch Oral Biol*. 2019 Apr;**100**:86-92. doi: 10.1016/j.archoralbio.2019.02.013. Epub 2019 Feb 20.

2. Pan S, Lei L, Chen S, Li H, Yan F. Rosiglitazone impedes Porphyromonas gingivalis-accelerated atherosclerosis by downregulating the TLR/NF-kappaB signaling pathway in atherosclerotic mice. *Int Immunopharmacol*. 2014;**23**(2):701-8. doi: 10.1016/j.intimp.2014.10.026.

3. Rivera MF, Lee JY, Aneja M, Goswami V, Liu L, Velsko IM, Chukkapalli SS, Bhattacharyya I, Chen H, Lucas AR, Kesavalu LN. Polymicrobial infection with major periodontal pathogens induced periodontal disease and aortic atherosclerosis in hyperlipidemic ApoE(null) mice. *PLoS One*. 2013;**8**(2):e57178. doi: 10.1371/journal.pone.0057178.

4. Nahid MA, Rivera M, Lucas A, Chan EKL, Kesavalu L. Polymicrobial Infection with Periodontal Pathogens Specifically Enhances MicroRNA miR-146a in ApoE(-/-) Mice during Experimental Periodontal Disease. *Infect Immun*. 2011;**79**(4):1597-605. doi: 10.1128/IAI.01062-10.

5. Poole S, Singhrao SK, Chukkapalli S, Rivera M, Velsko I, Kesavalu L, Crean S. Active invasion of Porphyromonas gingivalis and infection-induced complement activation in ApoE-/- mice brains. *J Alzheimers Dis*. 2015;**43**(1):67-80. doi: 10.3233/JAD-140315.

6. Velsko IM, Chukkapalli SS, Rivera-Kweh MF, Chen H, Zheng D, Bhattacharyya I, Gangula PR, Lucas AR, Kesavalu L. Fusobacterium nucleatum Alters Atherosclerosis Risk Factors and Enhances Inflammatory Markers with an Atheroprotective Immune Response in ApoE(null) Mice. *PLoS One*. 2015 Jun 16;**10**(6):e0129795. doi: 10.1371/journal.pone.0129795.

7. Chukkapalli SS, Rivera-Kweh MF, Velsko IM, Chen H, Zheng D, Bhattacharyya I, Gangula PR, Lucas AR, Kesavalu L. Chronic oral infection with major periodontal bacteria Tannerella forsythia modulates systemic atherosclerosis risk factors and inflammatory markers. *Pathog Dis*. 2015 Apr;**73**(3):ftv009. doi: 10.1093/femspd/ftv009.

8. Meyer RE, Fish RE. A review of tribromoethanol anesthesia for production of genetically engineered mice and rats. *Lab Anim (NY)*. 2005 Nov;**34**(10):47-52. doi: 10.1038/laban1105-47.

9. elsko IM, Chukkapalli SS, Rivera MF, Lee JY, Chen H, Zheng D, Bhattacharyya I, Gangula PR, Lucas AR, Kesavalu L. Active invasion of oral and aortic tissues by Porphyromonas gingivalis in mice causally links periodontitis and atherosclerosis. *PLoS One*. 2014 May 16;**9**(5):e97811. doi: 10.1371/journal.pone.0097811.

10. Rivera MF, Lee JY, Aneja M, Goswami V, Liu L, Velsko IM, Chukkapalli SS, Bhattacharyya I, Chen H, Lucas AR, Kesavalu LN. Polymicrobial infection with major periodontal pathogens induced periodontal disease and aortic atherosclerosis in hyperlipidemic ApoE(null) mice. *PLoS One.* 2013;**8**(2):e57178. doi: 10.1371/journal.pone.0057178.

11. Chukkapalli SS, Velsko IM, Rivera-Kweh MF, Zheng D, Lucas AR, Kesavalu L. Polymicrobial Oral Infection with Four Periodontal Bacteria Orchestrates a Distinct Inflammatory Response and Atherosclerosis in ApoE null Mice. *PLoS One*. 2015 Nov 30;**10**(11):e0143291. doi: 10.1371/journal.pone.0143291.

12. Lee C, Kim J, Shin SG, Hwang S. Absolute and relative QPCR quantification of plasmid copy number in Escherichia coli. *J Biotechnol*. 2006 May 29;**123**(3):273-80. doi: 10.1016/j.jbiotec.2005.11.014.

13. Canfrán-Duque A, Rotllan N, Zhang X, Fernández-Fuertes M, Ramírez-Hidalgo C, Araldi E, Daimiel L, Busto R, Fernández-Hernando C, Suárez Y. Macrophage deficiency of miR-21 promotes apoptosis, plaque necrosis, and vascular inflammation during atherogenesis. *EMBO Mol Med.* 2017 Sep;**9**(9):1244-1262. doi: 10.15252/emmm.201607492.

14. Dai J, Ma Y, Shi M, Cao Z, Zhang Y, Miron RJ. Initial changes in alveolar bone volume for sham-operated and ovariectomized rats in ligature-induced experimental periodontitis. *Clin Oral Investig.* 2016 Apr;**20**(3):581-8. doi: 10.1007/s00784-015-1531-3.

15. de Molon RS, Mascarenhas VI, de Avila ED, Finoti LS, Toffoli GB, Spolidorio DM, Scarel-Caminaga RM, Tetradis S, Cirelli JA. Long-term evaluation of oral gavage with periodontopathogens or ligature induction of experimental periodontal disease in mice. *Clin Oral Investig*. 2016 Jul;**20**(6):1203-16. doi: 10.1007/s00784-015-1607-0.

16. Kuo PJ, Fu E, Lin CY, Ku CT, Chiang CY, Fu MM, Fu MW, Tu HP, Chiu HC. Ameliorative effect of hesperidin on ligation-induced periodontitis in rats. *J Periodontol*. 2019 Mar;**90**(3):271-280. doi: 10.1002/JPER.16-0708.

17. Ulrich V, Rotllan N, Araldi E, Luciano A, Skroblin P, Abonnenc M, Perrotta P, Yin X, Bauer A, Leslie KL, Zhang P, Aryal B, Montgomery RL, Thum T, Martin K, Suarez Y, Mayr M, Fernandez-Hernando C, Sessa WC. Chronic miR-29 antagonism promotes favorable plaque remodeling in atherosclerotic mice. *EMBO Mol Med*. 2016 Jun 1;**8**(6):643-53. doi: 10.15252/emmm.201506031.

18. Seimon TA, Wang Y, Han S, Senokuchi T, Schrijvers DM, Kuriakose G, Tall AR, Tabas IA. Macrophage deficiency of p38alpha MAPK promotes apoptosis and plaque necrosis in advanced atherosclerotic lesions in mice. *J Clin Invest*. 2009 Apr;**119**(4):886-98. doi: 10.1172/JCI37262.

19. Liu L, Guo S, Shi W, Liu Q, Huo F, Wu Y, Tian W. Bone Marrow Mesenchymal Stem Cell-Derived Small Extracellular Vesicles Promote Periodontal Regeneration. *Tissue Eng Part A*. 2021 Jul;**27**(13-14):962-976. doi: 10.1089/ten.TEA.2020.0141.

20. Xian X, Ding Y, Dieckmann M, Zhou L, Plattner F, Liu M, Parks JS, Hammer RE, Boucher P, Tsai S, Herz J. LRP1 integrates murine macrophage cholesterol homeostasis and inflammatory responses in atherosclerosis. *Elife*. 2017 Nov 16;6:e29292. doi: 10.7554/eLife.29292.

21. Cai Y, Kurita-Ochiai T, Hashizume T, Yamamoto M. Green tea epigallocatechin-3-gallate attenuates Porphyromonas gingivalis-induced atherosclerosis. *Pathog Dis.* 2013 Feb;**67**(1):76-83. doi: 10.1111/2049-632X.12001.

22. Fukasawa A, Kurita-Ochiai T, Hashizume T, Kobayashi R, Akimoto Y, Yamamoto M. Porphyromonas gingivalis accelerates atherosclerosis in C57BL/6 mice fed a high-fat diet. *Immunopharmacol Immunotoxicol*. 2012 Jun;**34**(3):470-6. doi: 10.3109/08923973.2011.627866..

23. Crowley LC, Marfell BJ, Scott AP, Waterhouse NJ. Quantitation of Apoptosis and Necrosis by Annexin V Binding, Propidium Iodide Uptake, and Flow Cytometry. *Cold Spring Harb Protoc*. 2016 Nov 1;**2016**(11). doi: 10.1101/pdb.prot087288.

24. Vesterlund S, Paltta J, Karp M, Ouwehand AC. Measurement of bacterial adhesion-in vitro evaluation of different methods. *J Microbiol Methods.* 2005 Feb;**60**(2):225-33. doi: 10.1016/j.mimet.2004.09.013.

25. Sharma L, Wu W, Dholakiya SL, Gorasiya S, Wu J, Sitapara R, Patel V, Wang M, Zur M, Reddy S, Siegelaub N, Bamba K, Barile FA, Mantell LL. Assessment of phagocytic activity of cultured macrophages using fluorescence microscopy and flow cytometry. *Methods Mol Biol*. 2014;1172:137-45. doi: 10.1007/978-1-4939-0928-5_12.

26. Lehmann AK, Sornes S, Halstensen A. Phagocytosis: measurement by flow cytometry. *J Immunol Methods.* 2000 Sep 21;**243(**1-2):229-42. doi: 10.1016/s0022-1759(00)00237-4.

27. Li XY, Wang C, Xiang XR, Chen FC, Yang CM, Wu J. Porphyromonas gingivalis lipopolysaccharide increases lipid accumulation by affecting CD36 and ATP-binding cassette transporter A1 in macrophages. *Oncol Rep*. 2013 Sep;**30**(3):1329-36. doi: 10.3892/or.2013.2600. Epub 2013 Jul 8.

28. Xu S, Huang Y, Xie Y, Lan T, Le K, Chen J, Chen S, Gao S, Xu X, Shen X, Huang H, Liu P. Evaluation of foam cell formation in cultured macrophages: an improved method with Oil Red O staining and DiI-oxLDL uptake. *Cytotechnology*. 2010 Oct**;62**(5):473-81. doi: 10.1007/s10616-010-9290-0.

29. Ren Z, Yang Z, Lu Y, Zhang R, Yang H. Anti‑glycolipid disorder effect of epigallocatechin‑3‑gallate on high‑fat diet and STZ‑induced T2DM in mice. *Mol Med Rep*. 2020 Jun;**21**(6):2475-2483. doi: 10.3892/mmr.2020.11041.
